# Cu (II)-catalyzed: synthesis of imidazole derivatives and evaluating their larvicidal, antimicrobial activities with DFT and molecular docking studies

**DOI:** 10.1186/s13065-023-01067-1

**Published:** 2023-11-18

**Authors:** Janani Mullaivendhan, Idhayadhulla Akbar, Mansour K. Gatasheh, Ashraf Atef Hatamleh, Anis Ahamed, Mohamed Hussain Syed Abuthakir, Raman Gurusamy

**Affiliations:** 1 Research Department of Chemistry, Nehru Memorial College (Affiliated Bharathidasan University), Puthanampatti, 621007 Tamil Nadu India; 2 Department of Botany & Microbiology, College of Sciences, King Saud University (KSU), Riyadh, Saudi Arabia; 3 Institute of Systems Biology, Universiti Kebangsaan Malaysia (UKM), 43600 Bangi, Selangor Malaysia; 4 Department of Lifescience, Yeungnan University, Gyeondsan, Gyeondsan-Buk 38541 South Korea; 5 Department of Biochemistry, College of Science, King Saud University, P.O. Box 2455, Riyadh, 11451 Saudi Arabia

**Keywords:** Antibacterial, Antifungal, Larvicidal activity, DFT, Molecular docking, Mannich base

## Abstract

This paper deals with the evaluation of novel imidazole molecules for their antimicrobial and larvicidal activities. A series of imidazole derivatives **1(a**–**f)** and **2(a**–**e)** were prepared by the Mannich base technique using a Cu(II) catalyst. The Cu(phen)Cl_2 _catalyst was found to be more effective than other methods. FTIR, elemental analyses, mass spectrometry, ^1^H NMR, and ^13^C NMR spectroscopy were performed to elucidate the structures of the synthesised compounds. Antimicrobial and larvicidal activities were investigated for all compounds. The antibacterial activity of compounds (**2d**) and (**2a**) were highly active in *S.aureus* (MIC: 0.25 μg/mL) and *K.pneumoniae* (MIC: 0.25 μg/mL) compared to ciprofloxacin. Compound (**1c**) was significantly more effective than clotrimazole in *C.albicans* (MIC: 0.25 μg/mL). Molecular docking studies of compound **2d** showed a higher binding affinity for the 1BDD protein (− 3.4 kcal/mol) than ciprofloxacin (− 4.4 kcal/mol). Compound **1c** had a higher binding affinity (− 6.0 kcal/mol) than clotrimazole (− 3.1 kcal/mol) with greater frontier molecular orbital energy and reactivity properties of compound **1c** (∆E gap = 0.13 eV). The activity of compound **1a** (LD_50_: 34.9 μg/mL) was more effective in the *Culex quinquefasciatus* than permethrin (LD_50_: 35.4 μg/mL) and its molecular docking binding affinity for 3OGN protein (− 6.1 kcal/mol). These newly synthesised compounds can act as lead molecules for the development of larvicides and antibiotic agents.

**Supplementary Information:**

The online version contains supplementary material available at 10.1186/s13065-023-01067-1.

## Introduction

**Fig. 1:**
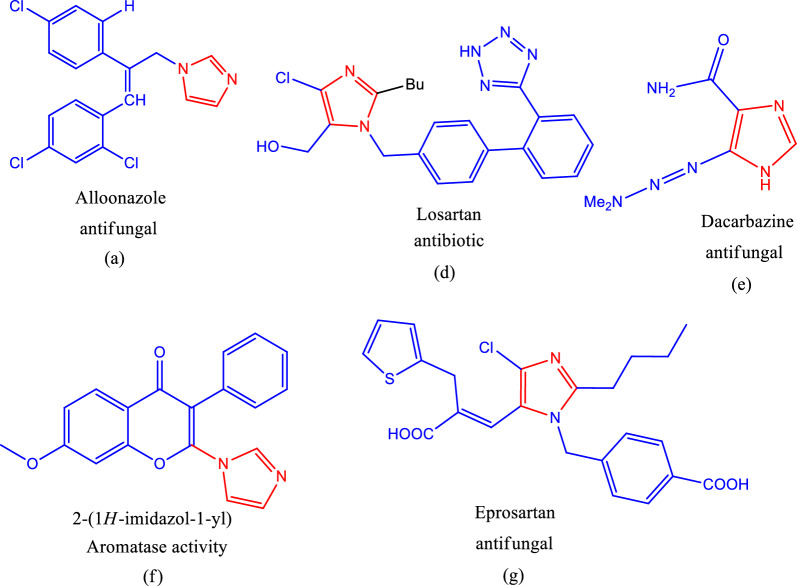
The Structure of typical of (1-methyl-1H-imidazole) based natural products

Important heterocyclic scaffolds, known as imidazoles, are used in a variety of applications in pharmaceuticals, natural products, endogenous chemicals, and polymers [1]. One of the most prized structures in medicinal chemistry is imidazole, and its derivatives display a variety of biological characteristics, including antidiabetic properties [2, 3]. It is also found in commercial drugs such as clotrimazole (antifungal), dipyrone (antipyretic), rimonabant (antiobesity), miconazole (antifungal), celecoxib (anti-inflammatory), clemizole (antihistaminic agent), (anti-inflammatory). Similarly, azoles are potent compounds with a vast range of therapeutic values, including antimicrobial [4, 5], anti-infective [6], anti-cancer [7], anti-tumor [8], anti-oxidant [9], and anti-viral [10, 11] activities. Azoles are well-known heterocyclic backbones owing to their drug-like properties and binding flexibilities. Naturally, azole derivatives such as pyrazole and imidazole are becoming increasingly important for drug development owing to their extensive biological activities. It is well established that numerous naturally occurring bioactive compounds that are part of this cycle have a wide range of pharmacological activities, including antibiotics [12], antifungals [13], anxiolytics [14], antivirals [15], and aromatase activity [16]. The biological activities of natural products are shown in (Fig. 1).

The urban mosquito laid eggs in standing water. Typically, in the Praires, the more common mosquito, the Aedes vexans, lays eggs in vegetation around water bodies, usually relying on large rainfall to hatch. Mosquitoes are among the deadliest arthropods in the world. They can act as vectors for various diseases and cause millions of deaths annually [17]. India has long struggled with serious public health issues related to the spread of mosquitoes. Therefore, it is imperative to control mosquitoes to prevent diseases such as West Nile virus infection, including malaria, chikungunya virus infection, yellow fever, lymphatic filariasis, Zika virus infection, and dengue fever, [18]. Various catalysts, such as copper (II) acetate [19], copper (II) acetylacetonate, dihydroxycopper [20], trifluoro methane sulfonate [21], dihydroxycopper, copper hydroxide phosphate, trifluoro methane sulfonate copper (II) [22], copper (II)pyridine, copper (II)chloride, and copper (II)iodide were synthesised through Mannich base derivatives in good yield, and as low yield was obtained in current studies so the dichloro-1,10-phenanthroline copper (II) catalyst was involved in this caltalyst optimisation process. Based on the above observations, this study aimed to synthesise new imidazole Mannich base derivatives using Cu(phen)Cl_2_ as a catalyst and investigate their antibacterial, antifungal, and larvicidal activities. Therefore, this study aimed to develop the best paradigm for Cu(II)-catalysed synthesis of imidazole derivatives of Mannich bases and to investigate their bioactivity. Since there are no detailed studies on the relationship between larvicidal and antimicrobial activities of imidazoles, the larvicidal and antibacterial effects of the compounds were assessed. The details of the new imidazole made by synthesising analogues and their larvicidal and antimicrobial activities with DFT and molecular docking studies are illustrated in the ongoing work.

## Material and methods

All chemicals are used as analytical grade and obtained from Sigma. FTIR (4000–400 cm^−1^) was used for Thermo Scientific Nicolet iS5. ^1^H and ^13^C NMR spectroscopy was used for Bruker DRX-300 MHz and 75 MHz spectrometer. Mass spectra were recorded by Clarus 690–SQ8MS (EI) from PerkinElmer GCMS. An elemental analyser (Model Varioel III) was used to calculate the concentrations of C, H, S, and N.

### Synthesis of compounds 1(a–f) and 2(a–e)

A mixture of l-histidine (0.1 mol), benzylidenehydrazine (0.1 mol), aldehyde (0.1 mol), and Cu(phen)Cl_2_ (10 mol%) was added to 30 ml of ethanol. The reaction mixture was refluxed for 3 h at 35 °C. The compound was identified by TLC using silica plates, and column chromatography was used to separate the final products. An average yield of 78–80% was obtained. All other compounds (**1b–f**), and (**2b–e**) were prepared using the same procedure.

### Optimization procedure for solvent and catalyst

Mannich base derivatives were prepared from the reactants, imidazole, benzylidene hydrazine, and para-substituted benzaldehyde. The reactions were performed under reflux at room temperature (35 °C) in the presence of Cu(phen)Cl_2_ catalysts in toluene, CH_2_Cl_2_, MeCN, H_2_O, EtOH, benzene, THF, and DMF. The reaction was carried out at 35 °C for 3 h. A variety of Cu(II)catalyst 10 mol% such as acetylacetonate, dihydroxy copper, copper hydroxide phosphate, trifluoromethanesulfonate, pyridine, dichloro-(1,10-phenanthroline) Cu(II), Cu(II)chloride, and Cu(I) iodide catalysts, have been used to synthesise compounds under reflux in EtOH at room temperature (35 °C) for 3 h.

### (2*S*)-2-Amino-3-(1-(((*E*)-2-benzylidenehydrazinyl)(phenyl)methyl)-1*H*-imidazol-4-yl)prop anoicacid (1a)

Yield 72%; Colour Pale yellow; mp 141–147 °C; R_f_ 0.66; IR(KBr): *ν*3385 (–NH), 3270 (–NH_2_), 2983 (–OH), 1730 cm^−1^; ^1^H NMR(DMSO-*d*_6_, 300 MHz): δ 11.3 (1H, –C=O–OH, s), 8.32 (–CH, 1H, s), 8.35–7.23 (10H, ph ring, m), 7.96, (N=CH–, 1H, s), 7.02 (1H, N–CH–, s),6.97 (1H, –NH–CH, s), 5.14 (2H, CH–NH_2,_ s), 4.16 (1H, C=O–CH, dd, *J* = 6 Hz, *J* = 9 Hz), 2.85 (2H, –CH_2_–, d, *J* = 6 Hz), 2.0 (1H, s, N–NH);^13^C NMR(DMSO-*d*_6_, 75 MHz): δ 174.9, 143.3, 138.6, 137.8, 136.4, 133.7, 131, 129.2, 128.8, 128.5, 126.9, 126.7, 118.8, 86.0, 55.1, 29.3: EI-MS (*m/z*): 364.15 (M^+^, 23.6%); Anal. calcd. for (C_20_H_21_NH_5_O_2_): C, 66.10; H, 5.82; N, 19.27; %; Found: C, 66.08; H, 5.81; N, 19.25%.

### (2*S*)-2-Amino-3-(1-(((*E*)-2-benzylidenehydrazinyl)(4-hydroxyphenyl)methyl)-1*H*-imidazol-4-yl)propanoic acid (1b)

Yield 79%; Colour Light brown; mp 140–142 °C; R_f_ 0.79; IR(KBr): *ν*3416 (–NH_2_), 3382 (–NH), 2857 (–OH), 1728 cm^−1^; ^1^H NMR(DMSO-*d*_6_, 300 MHz): δ 11.3 (–OH, 1H, s); 8.39 (1H, –CH, s), 8.35–7.51 (5H, Ar ring, m), 7.96 (N = CH–, 1H, s), 7.12–7.04 (4H, ph ring, d, *J* = 6 Hz), 7.03 (1H, N–CH–, s), 6.99 (1H, NH–CH, s), 5.35 (1H, Ph-OH, s), 5.11 (2H, CH-NH_2,_ s), 4.16 (1H, CH_2_-CH, dd, *J* = 6 Hz, *J* = 9 Hz), 2.83:2.80 (2H, -CH_2_-, d, *J* = 6 Hz), 2.0 (N–NH, 1H, s); ^13^C NMR(DMSO-*d*_6_, 75 MHz): δ 174.4, 156.5, 143.3, 137.8, 136.4, 133.7, 131.2, 131.0, 129.2, 128.8, 128.3, 118.8, 115.7, 86.0, 55.1, 29.3: EI-MS (*m/z*): 380.17 (M^+^, 22%); Anal. calcd. for (C_20_H_21_N_5_O_2_): C, 63.31; H, 5.58; N, 18.46%; Found: C, 63.29; H, 5.56; N, 18.43%.

### (2*S*)-2-Amino-3-(1-(((*E*)-2-benzylidenehydrazinyl)(4-chlorophenyl)methyl)-1*H*-imidazol-4-yl)propanoic acid (1c)

Yield 81%; Colour Pale yellow; mp 135–139 °C; R_f_ 0.34; IR(KBr): *ν*3398 (–NH), 3301 (–OH), 3274 (–NH_2_), 1725 cm^−1^; ^1^H NMR(DMSO-*d*_6_, 300 MHz): δ 11.3 (1H, –C=O–OH, s); 8.31 (–CH, 1H, s), 8.35–7.54 (5H, Ar ring, m), 7.96, (1H, N=CH–, s), 7.06–7.04 (ph ring, 4H, d, *J* = 6 Hz), 7.03 (N–CH–, 1H, s), 6.99 (1H, NH–CH, s), 5.14 (2H, CH–NH_2,_ s), 4.16 (1H, CH_2_–CH, dd, *J* = 6 Hz, *J* = 9 Hz), 2.83 (2H, d, *J* = 6 Hz, CH_2_–), 2.0 (N–NH, 1H, s); ^13^C NMR(DMSO-*d*_6_, 75 MHz): δ 174.7, 143.3, 137.8, 136.7, 136.4, 133.7, 132.3, 131.0, 129.2, 128.8, 128.6, 128.3, 118.8, 55.1, 29.3; EI-MS (*m/z*): 399.13 (M^+^, 32.8%); Anal. calcd. for (C_20_H_20_ClN_5_O_2_): C, 60.34; H, 5.09; N, 17.61%; Found: C, 60.36; H, 5.04; N, 17.58; %.

### (2*S*)-2-Amino-3-(1-(((*E*)-2-benzylidenehydrazinyl)(4-nitrophenyl)methyl)-1*H*-imidazol-4-yl)propanoic acid (1d)

Yield 83%; Colour White Solid; mp 167–171 °C; R_f_ 0.57; IR(KBr): *ν*3380 (–NH), 3327 (–OH), 3296 (–NH_2_), 1738 cm^−1^; ^1^H NMR(DMSO-*d*_6_, 300 MHz): δ 11.3 (–C=O–OH, 1H, s); 8.31 (1H, –CH, s); 8.35–7.52 (5H, Ar ring,m); 7.96, (N=CH–, 1H, s), 7.09–7.04 (ph ring, 4H, d, *J* = 6 Hz), 7.02 (N–CH–, 1H, s), 6.94 (1H, NH–CH, s), 5.11 (2H, CH–NH_2,_ s), 4.16 (1H, CH_2_–CH, dd, *J* = 6 Hz, *J* = 9 Hz), 2.83 (2H, –CH_2_–, d, *J* = 6 Hz), 2.2 (s, 1H, N–NH); ^13^C NMR(DMSO-*d*_6_, 75 MHz): δ 174.7, 145.9, 144.7, 143.3, 137.8, 136.4, 133.7, 131.0, 129.2, 128.8, 127.8, 123.7, 118.8, 86.0, 55.1, 29.3; EI-MS (*m/z*): 409.16 (M^+^, 22%); Anal. calcd. for (C_20_H_20_N_6_O_4_): C, 58.82; H, 4.94; N, 20.58%; Found: C, 58.80; H, 4.91; N, 20.56%.

### (2*S*)-2-Amino-3-(1-(((*E*)-2-benzylidenehydrazinyl)(4-methoxyphenyl)methyl)-1*H*-imidazol-4-yl)propanoic acid (1e)

Yield 78%; Colour Light brown; mp 176–182 °C; R_f_ 0.61; IR(KBr): *ν*3378 (–NH), 3302 (–NH_2_), 2844 (–OH), 1737 cm^−1^; ^1^H NMR(DMSO-*d*_6_, 300 MHz): δ 11.3 (–C=O–OH,1H, s), 8.36 (1H, –CH, s), 8.35–7.52 (5H, Ar ring, m), 7.96, (N=CH–, 1H, s), 7.06–7.04 (4H, ph ring, d, *J* = 6 Hz), 7.02 (N–CH–, 1H, s), 6.99 (1H, NH–CH, s), 5.11 (2H, CH–NH_2_, s), 4.16 (1H, CH_2_–CH, dd, *J* = 6 Hz, *J* = 9 Hz), 3.83 (3H, O–CH_3_, t), 2.80:2.83 (2H, –CH_2_–, d, *J* = 6 Hz), 2.1 (N–NH, 1H, s); ^13^C NMR(DMSO-*d*_6_, 75 MHz): δ 174.7, 158.8, 143.0, 137.8, 136.4, 133.7, 131.9, 131.0, 130.9, 129.2, 128.9, 127.9, 118.8, 114.1, 6.0, 55.8, 55.1, 29.3; EI-MS (*m/z*): 394.18 (M^+^, 24.7%); Anal. calcd. for (C_21_H_23_N_5_O_3)_: C, 64.11; H, 5.89; N, 17.80%; Found: C, 64.09; H, 5.86; N, 17.78%.

### (2*S*)-2-Amino-3-(1-(((*E*)-2-benzylidenehydrazinyl)(4-(dimethylamino)phenyl)methyl)-1*H*-imidazol-4-yl)propanoic acid (1f)

Yield 80%; Colour Pale yellow; mp 146–152 °C; R_f_ 0.62; IR(KBr): *ν*3380 (–NH), 3300 (–NH_2_), 3298 (–OH), 1729 cm^−1^; ^1^H NMR(DMSO-*d*_6_, 300 MHz): δ 11.3 (–C=O–OH, 1H, s), 8.39(–CH, 1H, s), 8.35–7.52 (5H, Ar ring, m), 7.96, (1H, N=CH–, s), 7.05–7.02 (4H, Ph ring, d, *J* = 3 Hz), 7.02 (1H, –N–CH–, d, *J* = 3 Hz), 6.94 (NH–CH, 1H, s), 5.11 (CH–NH_2,_ 2H, s), 4.16 (CH_2_–CH, 1H, dd, *J* = 6 Hz, *J* = 9 Hz),2.83 (d, 2H, *J* = 6 Hz, –CH_2_–), 3.06 (6H, N–(CH_3_)_2,_ s), 2.1 (N–NH, 1H, s); ^13^C NMR(DMSO-*d*_6_, 75 MHz): δ 174.7, 149.0, 143.4, 137.8, 136.4, 133.7, 131.0, 129.2, 128.8, 128.1, 127.8, 118.8, 112.7, 86.0, 55.1, 41.3, 29.3; EI-MS (*m/z*): 407.22 (M^+^, 24.2%); Anal. calcd. for (C_22_H_26_N_6_O): C, 65.04; H, 6.47; N, 20.65%; Found: C, 65.00; H, 6.43; N, 20.66%.

### (2S)-2-Amino-3-(1-((E)-1-((E)-2-benzylidenehydrazinyl)-3,7-dimethylocta-2,6-dien-1-yl)-1H-imidazol-4-yl)propanoic acid (2a)

Yield 73%; Colour Light brown; mp 158–160 °C; R_f_ 0.42; IR(KBr):* ν*3375 (–NH), 3296 (–NH_2_), 3081 (–OH), 1744 cm^−1^; ^1^H NMR(DMSO-*d*_6_, 300 MHz): δ 11.5 (–OH, 1H, s), 8.36 (–CH, 1H, s), 8.35–7.52 (5H, Ar ring, m), 7.83 (–NH, 1H, s), 6.88 (–CH, 1H, s), 6.64 (–N–C, 1H, s), 5.31 (1H, s), 5.18 (1H, –CH, s), 5.11 (2H, –OH, s), 4.16 (1H, CH_2_–CH, dd, *J* = 6 Hz, *J* = 9 Hz), 2.83 (–CH_2_–, d, *J* = 6 Hz, 2H), 2.18 (2H, –CH_2,_ s), 2.2 (1H, –NH, s), 1.98 (2H, –CH_2,_ s), 1.85 (3H, s, –C–CH_3_), 1.81 (–CH_3,_ 3H, s), 1.68 (–C–CH_3,_ 3H, s); ^13^C NMR(DMSO-*d*_6_, 75 MHz): δ 174.4, 143.3, 137.8, 136.4, 136.2, 135.5, 133.7, 132.0, 131.4, 131.0, 129.2, 128.8, 123.5, 118.8, 118.1, 79.3, 55.1, 39.4, 29.3, 27.6, 24.6, 18.6, 16.1; EI-MS (*m/z*): 424.27 (M^+^, 26.4%); Anal. calcd. for (C_24_H_31_N_5_O_2_): C, 67.46; H, 7.63; N, 17.10%; Found: C, 67.45; H, 7.60; N, 17.11%.

### (2*S*)-2-Amino-3-(1-(1-((*E*)-2-benzylidenehydrazinyl)-3-methylbut-2-en-1-yl)-1*H*-imidazol-4-yl)propanoic acid (2b)

Yield 76%; Colour Light brown; mp 163–165 °C; R_f_ 0.47; IR(KBr): *ν*3350 (–NH), 3297 (–NH_2_), 2837 (–OH), 1746 cm^−1^; ^1^H NMR(DMSO-*d*_6_, 300 MHz): δ 11.5 (–OH, 1H, s), 8.36 ( s, 1H, –CH), 8.35–7.52 (5H, Ar ring, m), 7.84 (–NH, 1H, s), 6.88 (1H, –CH, s), 6.63 (s, 1H, –N–C), 5.33 (1H, –H, s), 5.11 (2H, –NH_2,_ s), 4.16 (1H, CH_2_–CH, dd, *J* = 6 Hz, *J* = 9 Hz), 2.80;2.83 (2H, d, *J* = 6 Hz, –CH_2_–), 2.0 (1H, –NH, s), 1.82 (–CH_3,_3H, s), 1.68 (s, –CH_3,_ 3H); ^13^C NMR(DMSO-*d*_6_, 75 MHz): δ 174.7, 143.3, 137.8, 136.4, 133.7, 131.8, 131.0, 129.2, 128.8, 119.5, 118.8, 79.0, 55.1, 24.3, 18.3; EI-MS (*m/z*): 342.19 (M^+^, 19.8%); Anal. calcd. for (C_18_H_23_N_5_O_2_): C, 63.35; H, 6.76; N, 20.53%; Found: C, 63.38; H, 6.76; N, 20.49%.

### (2*S*)-2-Amino-3-(1-(((*E*)-2-benzylidenehydrazinyl)(furan-2-yl)methyl)-*1H*-imidazol-4-yl)propanoic acid (2c)

Yield 81%; Colour Light yellow; mp 178–181 °C; R_f_ 0.53; IR(KBr): *ν*3395 (–NH), 3300 (–NH_2_), 2936 (–OH), 1742 cm^−1^; ^1^H NMR(DMSO-*d*_6_, 300 MHz): δ 11.5 (–OH, 1H, s), 8.37 (–CH, 1H, s), 8.35–7.52 (5H, Ar ring, m), 7.83 (1H, –NH, s), 7.62–7.64 (3H, Furan, dd, *J* = 6 Hz, *J* = 9 Hz),6.87(1H, –CH, s), 6.62 (1H, –N–C, s), 5.11 (2H, –OH, s), 4.16 (1H, CH_2_–CH, dd, *J* = 6 Hz, *J* = 9 Hz),2.83 (2H, –CH_2_–, d, *J* = 6 Hz), 2.1 (1H, s, –NH); ^13^C NMR(DMSO-*d*_6_, 75 MHz): δ 174.9, 152.5, 143.3, 142.1, 137.8, 136.4, 133.7, 131.0, 129.2, 128.8, 118.8, 110.6, 106.7, 87.2, 55.1, 29.3; EI-MS (*m/z*): 354.15 (M^+^, 21.4%); Anal. calcd. for (C_18_H_19_N_5_O_3_): C, 61.19; H, 5.40; N, 19.85%; Found: C, 61.16; H, 5.40; N, 19.81%.

### (2*S*)-2-Amino-3-(1-(((*E*)-2-benzylidenehydrazinyl)(pyridin-4-yl)methyl)-1*H*-imidazol-4-yl)propanoic acid (2d)

Yield 83%; Colour White Solid; mp 145–149 °C; R_f_ 0.61; IR(KBr): *ν*3385 (–NH), 3324 (–OH), 3285 (–NH_2_), 1744 cm^−1^; ^1^H NMR(DMSO-*d*_6_, 300 MHz): δ 11.3 (1H, –OH, s), 8.54–8.50 (4H, pyridine, d, *J* = 6 Hz), 8.34 (s, 1H, –CH), 7.88–7.83 (Ar ring, m, 5H), 7.84 (–NH, 1H, s), 6.88 (s, –CH, 1H), 6.63 (1H, –N–C, s), 5.11 (2H, –OH, s), 4.16 (2H, CH_2_–CH, dd, *J* = 6 Hz, *J* = 9 Hz), 2.83 (d, *J* = 6 Hz, –CH_2_–, 2H), 2.3 (1H, s, –NH); ^13^C NMR(DMSO-*d*_6_, 75 MHz): δ 174.7, 149.8, 146.5, 143.3, 137.8, 136.4, 133.7, 131.0, 129.2, 128.8, 124.2, 118.8, 86.0, 55.1, 29.3; EI-MS (*m/z*): 365.17 (M^+^, 20.9%); Anal. calcd. for (C_19_H_20_N_6_O_2_): C, 62.62; H, 5.53; N, 23.06%; Found: C, 62.59; H, 5.50; N, 23.04%.

### (2*S*)-2-Amino-3-(1-(((*E*)-2-benzylidenehydrazinyl)-3-phenylallyl)-1*H*-imidazol-4- yl)propanoic acid (2e)

Yield 80%; Colour White Solid; mp153–159 °C; R_f_ 0.29; IR(KBr): *ν*3340 (–NH), 3295 (–NH_2_), 3091 (–OH), 1740 cm^−1^; ^1^H NMR (DMSO-*d*_6_, 300 MHz): δ 11.2 (–OH, 1H, s), 8.36 (s, 1H, ph–CH–), 8.35–7.52 (5H, Ar ring, m), 7.83 (1H, –NH,s), 7.38–7.24 (5H, Ar ring, m), 6.88 (1H, –CH, s), 6.62 (1H, –N–C, s), 6.56–6.19 (2H, C–H, s), 5.11 (2H, –OH, s), 4.19 (1H, d, *J* = 6 Hz, d, *J* = 9 Hz,–CH_2_–CH), 2.84 (2H, d, *J* = 6 Hz,–CH_2_–), 2.3 (–NH, 1H, s); ^13^C NMR (DMSO-*d*_6_, 75 MHz): δ 174.9, 137.6, 136.3, 133.7, 129.5, 129.2, 128.8, 128.6, 128.5, 127.9, 123.3, 118.8, 85.4, 55.1, 29.3; EI-MS (*m/z*): 390.19 (M^+^, 24.1%); Anal. calcd. for (C_22_H_23_N_5_O_2_): C, 67.83; H, 5.98; N, 17.96%; Found: C, 67.83; H, 5.92; N, 17.96%.

### Biological screening

#### Microorganisms

The Microbial Type Culture Collection Centre, Institute of Microbial Technology, Chandigarh, India, provides the varies microorganisms. All test microorganisms were kept alive on nutritional agar slants (HiMedia) maintained at 4 °C. The assay was performed using disk diffusion and broth dilution methods. *Staphylococcus aureus* (MTCC 96), *Escherichia coli* (MTCC 739), *Pseudomononas aeruginosa* (MTCC 2453), *Klebsiella pneumoniae* (MTCC 109), and were used for the antibacterial test. Antifungal tests were performed in *Candida albicans* (MTCC 183), *Microsporum audouinii* (MTCC 739), *Cryptococcus neoformans* (a clinical isolate), and *Aspergillus niger* (MTCC 872). Fresh cultures of each microbe a loop containing the were formed by transferring stock culture inoculum into test tubes containing autoclaved nutrient broth.

#### In vitro antibacterial screening

Compounds **1(a**–**f)** and **2(a**–**e)** were tested in *S. aureus, E. coli, P.aeruginosa,* and *K. pneumoniae*. Bacterial inocula were prepared from fresh overnight cultures, suspended in 0.85% saline, and adjusted to a McFarland turbidity of 0.5. Mueller–Hinton agar (HiMedia, India) was uniformly streaked over the suspension. A sterile cork borer was used to create a well measuring five millimeters in diameter, which was filled with 100 µL of the test compound (100 μg/mL). The positive control was ciprofloxacin and the negative control was DMSO. The plates were incubated at 37 °C for 24 h. Three sets of tests were conducted to validate the findings statistically.

#### In vitro antifungal screening

Antifungal activity in *C. albicans*, *C. neoformans*, and *M. audouiniiwas* evaluated for compounds **1(a–f),** and **2(a–e)** using the method described above. Positive and negative controls were used to validate the inferences.

### Determining the minimal inhibitory concentration (MIC)

Compounds **1 (a**–**f)** and **2 (a**–**e)** were dissolved in 64 μg/mL DMSO. The solutions at 64, 32, 16, 8, 4, 2, 1, 0.5, and 0.25 μg/mL were made using a twofold dilution. In each well, 106 colonies of microbes per millilitre (unit/mL) of suspension were cultured for 24 h at 37 °C. The minimal inhibitory concentrations of compounds with no noticeable growth were identified.

### Larvicidal activity

The susceptibility of *C. quinquefasciatus* to compounds **1(a**–**f)** and **2(a**–**e)** was determined using a standard bioassay protocol as described in our previous work. The 2 and 3 stage larvae (ten/vial) were placed in a test vial. Mortality was checked using various concentrations (10, 25, 50, and 100 μg/mL) of the synthesised compounds **1(a–f), 2(a–e),** and positive (DMSO) and negative (without vehicle) controls after a 24 h exposure period, and the number of surviving larvae was recorded. To verify the outcomes, each experiment was performed three times.

### Molecular docking analysis

To identify the mode of interaction, molecular docking experiments were completed, and the binding of the most potent molecules in the imidazole series (**2d, 1c,** and **1a**) and proteins 1BDD, 1AI9, and 3OGN were assessed using AutoDock Vina 1.1.2. The highly active compounds in the molecular docking models were compared with standard drugs such as ciprofloxacin, clotrimazole, and permethrin.

## Results and discussion

### Chemistry

**Scheme 1:**
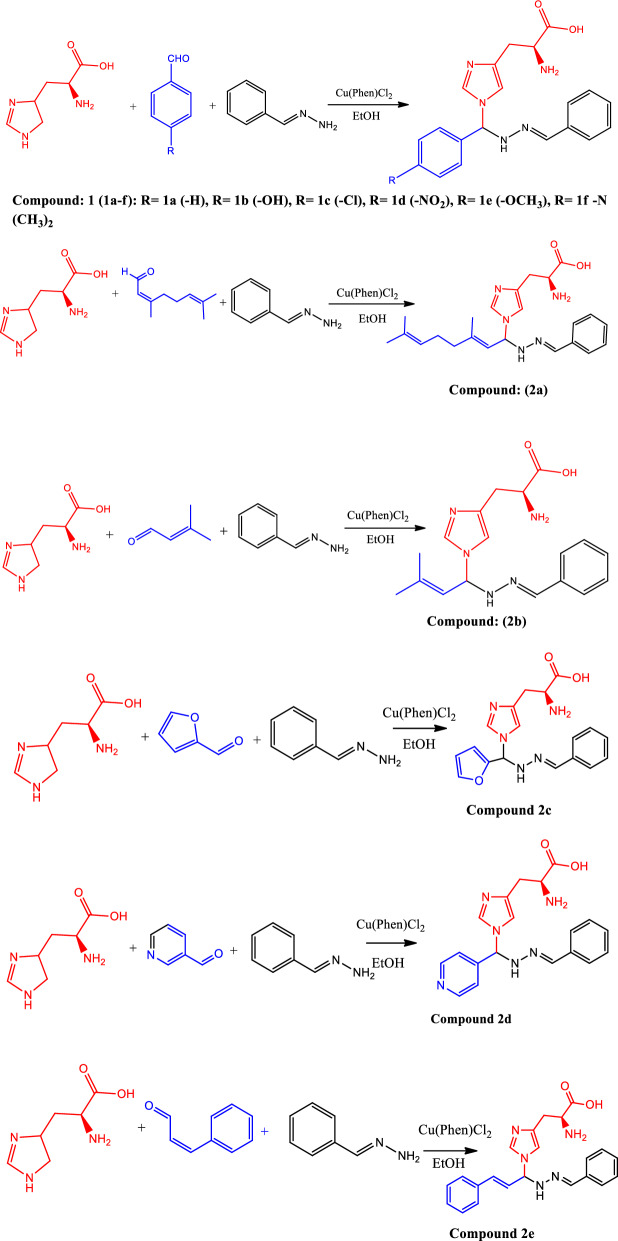
The synthetic route of compounds (1a-f) and (2a-e)

The three-component reactions of l-histidine, benzylidenehydrazine, and aldehydes were carried out using a conversion method to create Mannich-based imidazole derivatives. The reaction sequence is showed in Scheme 1. Various solvents such as MeCN, THF, toluene, CH_2_Cl_2_, EtOH, benzene, H_2_O, and DMF, and various Cu(II) catalysts were used to optimise the reaction for **1a**. The Cu(phen)Cl_2_ catalyst gave an excellent yield compared to other Cu(II) catalysts. The Cu(phen)Cl_2_ catalyst was produced in higher yields for compound **1a** in ethanol solvent (Table 1, entry 7). Under optimum conditions, imidazole, benzylidene hydrazine, and Cu(II) catalysts using different aldehydes together with para-substituted benzaldehyde produced imidazole derivatives **1(a–f)** and **2(a–e)** in good yields. Using 10 mol% Cu(phen)Cl_2_ in ethanol, the target product **1a** was prepared with dichloro-(1,10-phenanthroline)-copper (II) in 92% yield within 3 h. The high catalytic activity of **1a** is summarised in Table 2.

**Table 1:** The compound 1a from different solvent with Cu(Phen)Cl_2_ catalyst

Entry	Solvent	Yield
1	Toluene	No reaction
2	CH_2_Cl_2_	48
3	MeCN	77
4	H_2_O	37
5	EtOH	92
6	Benzene	No reaction
7	THF	No reaction
8	DMF	No reaction

All reaction were carried out at r.t for 3 h

**Table 2:** The compound 1a synthesized from ethanol solvent with different Cu(II) catalyst

Entry	Catalyst	Yield %
1	Copper (II)acetate	65
2	Copper (II) acetylacetonate	58
3	Dihydroxycopper	49
4	Copperhydroxide phosphate	68
5	Trifluoromethanesulfonate Copper(II)	52
6	Trifluoromethanesulfonate Copper(II)pyridine	61
7	Dichloro(1,10-phenanthroline)copper(II)	92
8	Copper(II)chloride	57
9	Copper(I) iodide	44

All reactions were carried out with 10 mol % of catalyst for 3 h in EtOH at r.t

The infrared spectra of all compounds were observed at 3398–3375, 3298–2837, 3416–3270, and 1750–1730 cm^−1^, corresponding to the –C=O, –NH, –OH, and –NH_2_ groups, respectively. Compound **1a**'s ^1^H NMR spectrum reveals that its chemical shift of **1a** was 118.1 ppm. This corresponds to the proton OH bound to the carbonyl group, a singlet in this region, while δ 8.36 corresponds to the proton Ph- CH (d, *J* = 3 Hz) with a singlet. The chemical shift of δ 7.96 corresponds to the –N=CH– proton in a singlet. The chemical shift δ 7.83–7.26 corresponds to the 5H protons in the phenyl ring representing mutilate in this reignite. A chemical shift of δ 7.02 corresponds to singlets in the region, which is represented by the –N–CH– group. Another chemical shift, δ 6.11, corresponds to the proton –NH–CH– means, a singlet in this region. The chemical shift δ 5.13 corresponds to the 2H proton in the –CH–NH_2_ group, indicating this region's singlet. The chemical shift δ 4.16 (dd, *J* = 6 Hz, *J* = 9 Hz) corresponded to the 1H proton in the –CO–CH–group, which correlated with the singlet in this region. The chemical shift δ 3.11 (d, *J* = 6 Hz) corresponded to the 1H proton in the –CH–group, which coincided with the singlet in this region. The chemical shift δ 2.86 corresponds to the 1H proton in the –CH– group and δ 2.0 was observed the 1H proton in the –N–NH group, which matched the singlet in this region. The common chemical shift values of δ 8.3–7.96, 11.0–5.11 and 5.13–4.18 correspond to the –N = CH, –OH, and –NH_2_ protons, respectively, present in all the synthesised compounds 1(**b–f**) and 2(**a–e**).

The ^13^C NMR chemical shift value of compound **1a** showed that the signals at δ 174.7 corresponded to the –C = O of the carbon present in the carboxyl group. The chemical shift value of δ 143.3 corresponds to the –CH group presence in compound **1a,** the δ 138.6–126.9 corresponds to the present in the aromatic ring, the δ 137.8–118.8 corresponding to the present in the imidazole, the value of δ 133.7–128.8 representing the in the aromatic ring, the value of δ 86.0 representing the –CH– presence. The values of δ 55.1 and 29.3 correspond to –CH– and –CH_2_– carbons, respectively. The common chemical shift values of δ 174.7, 143.3–137.8, 137.8–118.6, and 55.1 ppm corresponded to the –CO, –C = C, –C = N and –C–NH_2_ groups present in all synthesised compounds **1(b–f)** and **2(a–e),** respectively. Mass spectrometry was used to determine the molecular weight of **1a,** which showed that the molecular ion peak corresponded to EI-MS (m/z):364.15 (M^ +^ , 10%). The structures of the components were verified by mass spectroscopy and elemental analysis. Compounds **1(b–f)** and **2(a–e)** were characterised following the method described above for compound **1a**. FTIR, NMR, and mass spectraum (Additional file 1: Fig. S1–S42) are presented, and the ^1^H and ^13^C NMR values are tabulated and presented in Supporting Information (Additional file 1: Table S1–11 and Fig. S43–53).

### Biological activities

#### Antibacterial activity

Compounds **1(a–f)** and **2(a–e)** were evaluated for their antibacterial activities in both gram-positive and gram-negative bacteria [23]. Compound **2d** was more active in *S. aureus* (MIC: 0.25 μg/mL) than ciprofloxacin (MIC: 0.5 μg/mL). Compound **2a** showed higher activity in *K. pneumonia* (MIC: 0.25 μg/mL) than ciprofloxacin (MIC:32 μg/mL). In contrast, compounds **1a, 1b, 1c, 1d, 1e,** and **2b** showed lower activity in all bacterial strains than that of the ciprofloxacin standard (MIC: 32 μg/mL). Compound **2c** showed similar activity in *S. aureus* (MIC: 0.5 μg/mL) compared to ciprofloxacin (MIC:0.5 μg/mL). In addition, it was highly active in *K. pneumoniae* (MIC: 0.5 μg/mL). Compound **1e** exhibited equipment activity in *K. pneumoniae* (MIC: 32 μg/mL) compared to standard. These values are presented in Tables 3 and 5, respectively.

**Table 3:** Compounds **1 (a**–**f), 2 (a**–**e),** and zone of inhibition/mm's antibacterial activity

Compounds	Gram positive		Gram negative	
	*Escherichia Coli*	*Staphylococcus Aureus*	*Klebsiella Pneumoniae*	*Pseudomononas Aeruginosa*
**1a**	10	12	15	18
**1b**	05	10	10	12
**1c**	14	08	13	14
**1d**	12	05	10	12
**1e**	10	10	18	10
**1f**	09	12	20	22
**2a**	10	15	26	21
**2b**	10	17	12	10
**2c**	16	20	20	22
**2d**	15	25	18	10
**2e**	17	10	20	18
**DMSO**	–	–	–	–
**Ciprofloxacin**	16	22	16	25

(–) nil active

#### Antifungal activity

The antifungal activities of compounds **1(a–f)** and **2(a–e)** were evaluated using the disc diffusion method [24] in *C. neoformans, C. albicans, and M. audouinii* fungal strains. Compound **1c** was more effective in *C. albicans* (MIC: 0.25 μg/mL) than cotrimazole (MIC:0.5 g/mL). Compound **1b** was highly activity in *C. albicans* (MIC:0.25 μg/mL) than clotrimazole. Compounds **1b, 2a,** and **2b** were more active in *A. niger* (MIC: 16 μg/mL) than clotrimazole. Compounds **1a** and **2d** showed equipment activity (MIC: 16 μg/mL) in *Cryptococcus neoformans* compared to clotrimazole. These values are accessible in Tables 4 and 5, respectively.

**Table 4:** Compounds **1 (a**–**f),** and **2 (a**–**e),** zone of inhibition/mm's antifungal activity

Compound	*Aspergillus niger*	*Candida albicans*	*Microsporum audouinii*	*Cryptococcus Neoformans*
**1a**	16	20	10	14
**1b**	18	22	12	10
**1c**	17	26	10	12
**1d**	14	20	13	16
**1e**	12	10	10	12
**1f**	10	05	18	20
**2a**	15	25	18	10
**2b**	17	10	20	18
**2c**	12	10	22	20
**2d**	10	12	16	18
**2e**	15	25	18	10
**DMSO**	–	–	–	–
**Clotrimazole**	20	24	15	20

(–) nil active

**Table 5:** Compounds **1(a**–**f), 2(a**–**e),** minimal inhibitory concentrations

Comp. No	Minimum inhibitory concentration (MIC)/μg/Ml							
	Antibacterial activity				Antifungal activity			
	*E. C*	*S. a*	*K. p*	*P. a*	*A. n*	*C. a*	*M. a*	*Cr. n*
**1a**	> 100	64	64	32	32	16	> 100	16
**1b**	> 100	> 100	> 100	64	16	0.5	64	> 100
**1c**	32	> 100	64	32	32	0.25	> 100	64
**1d**	64	> 100	> 100	64	32	16	64	32
**1e**	> 100	> 100	32	> 100	64	> 100	> 100	62
**1f**	> 100	64	16	0.5	> 100	> 100	32	62
**2a**	> 100	32	0.25	32	16	32	16	> 100
**2b**	> 100	16	64	> 100	16	> 100	16	62
**2c**	32	0.5	0.5	0.5	64	> 100	32	62
**2d**	32	0.25	16	> 100	> 100	> 100	32	16
**2e**	16	> 100	16	16	32	16	32	> 100
**DMSO**	–	–	–	–	–	–	–	–
**Ciprofloxacin**	0.5	0.5	32	0.25	–	–	–	–
**Clotrimazole**	–	–	–	–	32	0.5	08	32

(–) nil active

#### Larvicidal activity

The larvae of the second instar of *C. quinquefasciatus* were used for larvicidal screening [25] of all synthesised compounds **1(a–f)** and **2(a–e)**. Compound 1a showed higher activity (LD_50_: 34.9 μg/mL) than other compounds and permethrin (LD_50_: 35.4 μg/mL). Compounds **1c, 2a,** and **2e** showed nearly equipotent activity compared to that of permethrin. Compound **2c** had an LD_50_ value greater than or equal to 100 μg/mL, indicating its low activity in *C. quinquefasciatus*. The values are listed in Table 6.

**Table 6:** Larvicidal profiles of compounds **1(a–f), 2(a–e)** on Culex sp. second-instar larvae

Compound	Mortality (%)^a^				LD_50_ (μg/mL)
	Concentration (μg/mL)				
	10	25	50	100	
**1a**	15.2 ± 0.31	53.1 ± 0.31	80.2 ± 0.34	91.3 ± 0.34	34.9
**1b**	12.3 ± 0.39	22.3 ± 0.39	79.5 ± 0.34	82.3 ± 0.34	47.3
**1c**	19.2 ± 0.31	35.2 ± 0.31	76.2 ± 0.34	90.3 ± 0.34	39.6
**1d**	12.3 ± 0.39	22.3 ± 0.39	79.5 ± 0.34	82.3 ± 0.34	47.3
**1e**	15.2 ± 0.31	25.2 ± 0.31	86.2 ± 0.34	92.3 ± 0.34	40.9
**1f**	12.3 ± 0.39	32.3 ± 0.39	79.5 ± 0.34	82.3 ± 0.34	44.1
**2a**	15.2 ± 0.31	35.2 ± 0.31	88.2 ± 0.34	95.3 ± 0.34	36.7
**2b**	12.3 ± 0.39	42.3 ± 0.39	79.5 ± 0.34	82.3 ± 0.34	40.5
**2c**	–	–	15.2 ± 0.31	25.2 ± 0.31	< 100
**2d**	12.3 ± 0.39	22.3 ± 0.39	79.5 ± 0.34	82.3 ± 0.34	47.3
**2e**	15.2 ± 0.31	35.2 ± 0.31	86.2 ± 0.34	92.3 ± 0.34	37.7
**DMSO**	–	–	–	–	–
**Permethrin**	11.1 ± 0.19	51.1 ± 0.19	76.3 ± 0.14	100 ± 0.0	35.4

(–) nil active,The values represented the three-replicate ± SD

### Structure–activity relationship

**Fig. 2:**
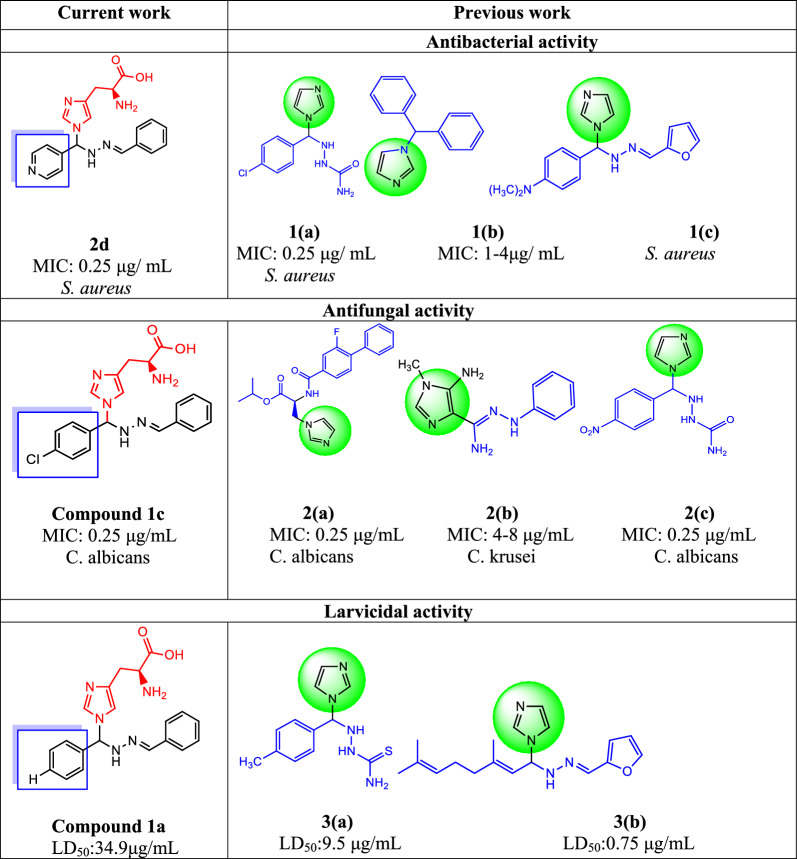
Previous and current of structure–activity relationship

The synthesised compounds **1(a–f)** and **2(a–e)** were examined for their relationship with structure and activity. Compounds **1a, 1b, 1c, 2a, 2c, 2d,** and **2e** were particularly active. Figure 2 shows the structure–activity relationship.

Compound **1(a)** have para -Cl in phenyl ring, which is highly active in *S. aureus* (MIC:0.25 μg/mL) than ciprofloxacin (MIC:0.5 μg/mL) [26]. Compound **1b**, an imidazole moiety, showed the most effective antibacterial action because of its small size and improved ability to enter the bacterial cells. Compound **1b**'s antibacterial activity was reduced by chlorine at the para position of the phenyl ring on the imidazole derivatives (MIC: 1–4 g/mL) [27]. This area plays a biological role in imidazole and the para-substituted phenyl ring. In antifungal screening, compound **1c**, which has an –N(CH_3_)_2_ group in the phenyl ring, showed higher activity in *C. neoformans* and *S. aureus* [28]. In comparison with ciprofloxacin (pyridine-3-carboxylic acid), compound **2d** (pyridine ring moiety with imidazole moiety and 2-amino acetic acid) (MIC: 0.25 g/mL) showed significantly higher activity in *S. aureus.* Similar to ciprofloxacin, compound **2d** showed high activity in *K. pneumoniae* (MIC: 0.25 μg/mL). Compound **2c**, which contains a furan and imidazole moiety with 2-amino acetic acid, exhibits higher action in *S. aureus* and *K. pneumonia* (MIC: 0.5 μg/mL) in comparison to standard ciprofloxacin (MIC: 0.5 μg/mL). In addition, compound **1e** (anisole connected with an imidazole moiety and 2-amino acetic acid) exhibited equipotential activity (MIC: 32 μg/mL) in *K. pneumoniae* compared to ciprofloxacin. Antibacterial and antifungal activities have been demonstrated in recent studies. The biphenyl carboxamide connected to an imidazole moiety was able to create essential antifungal agents and fluconazole (MIC: 2–8 μg/mL), highly active *C. tropicalis*, and *C. albicans* (MIC:0.5 μg/mL). However, halogen groups in the ortho or para positions of the aromatic ring increase the effects because they act as electron-donating groups [29]. Previous studies have aimed to clarify the mechanism through which 5-aminoimidazole-4-carbohydrazonamide derivatives act as antifungal agents in *C. albicans* (MIC: 32–64 μg/mL) and *C. krusei* (MIC: 4–8 μg/mL) compound **2(b)** with fluconazole. The relationship with this antifungal medication results from the suppression of the *C. albicans* virulence mechanism, which is a dimorphic transition [30]. Compound **2(c),** which has a para-NO_2_ group, demonstrated effective antifungal activity in *C. albicans*(MIC: 0.25 μg/mL) than other synthesised compounds and clotrimazole (MIC:1 μg/mL) [26]. Compound **1a**, which contains an imidazole moiety with 2-amino acetic acid, and **1c**, which contains para-chlorophenyl and an imidazole moiety with 2-amino acetic acid, **1d,** and **2c,** which contain a furan and an imidazole moiety with 2-amino acetic acid, were all equally effective in *A. niger* and clotrimazole. Compounds **1b, 2a,** and **2b** were more active in *A. niger* than the clotrimazole. Compound **1c** (4-chloro phenyl and imidazole moiety with 2-amino acetic acid) was highly active in *C. albicans* (MIC: 0.25 μg/mL) than standard clotrimazole (MIC: 0.5 μg/mL). Clotrimazole (MIC: 0.5 μg/mL) compared to compound **1b** (4-hydroxyl phenyl and imidazole moiety with 2-amino acetic acid) showed more equipment activity. Compounds **1a** (imidazole moiety with 2-amino acetic acid) and **2d** showed equipotent activity (MIC: 16 μg/mL), whereas compound **1d** (pyridine ring and imidazole moiety with 2-amino acetic acid) had equipotent activity (MIC: 16 μg/mL) compared than clotrimazole (MIC: 16 μg/mL). Previous and present studies of larvicidal activity: As an effective larvicide (LD_50_: 9.5 μg/mL) due to compound **3(a)** containing a para-CH_3_-phenyl, thiosemicarbazone and imidazole ring [26]. larvicidal screening, compound **3(b)**, which has a 2,6-dimethylocta-2,6-diene group, is highly toxic (LD_50_: 0.75 μg/mL) compared to other compounds [28]. Compared to permethrin, compound **1a** (imidazole moiety with 2-amino acetic acid) showed higher larvicidal activity (LD_50_: 34.9 μg/mL). When compared to permethrin, the activities of compounds **1c** (4-chloro phenyl and imidazole moiety with 2-amino acetic acid), **2a** (citral connected with imidazole moiety with 2-amino acetic acid), and **2e** (cinnamaldehyde connected with imidazole moiety with 2-amino acetic acid) were virtually equal to LD_50_values more than or equal to 100 μg/mL, while compound **2c** (furan and imidazole moiety with 2-amino acetic acid) exhibited low activity in *C. quinquefasciatus*. The above pieces of evidence indicate the relationship between the antibacterial, antifungal, and larvicidal activities of previous and current studies, as shown in (Fig. 2).

### Molecular docking studies with auto dock vina

The processes of adsorption and interactions among the most potent molecules in the imidazole series (i.e. **2d, 1c,** and **1a**) and proteins 1BDD, 1A19, and 3OGN were investigated using molecular docking studies with AutoDock Vina 1.1.2. The outcomes were evaluated using reference molecular docking models. Ciprofloxacin, clotrimazole, and permethrin were used to compare molecular docking studies. *Staphylococcus aureus* protein a (PDB ID:1BDD), Candida albicans (PDB ID :1AI9), and odourant-binding protein (PDB ID:3OGN) were obtained from the Protein Data Bank.

**Fig. 3:**
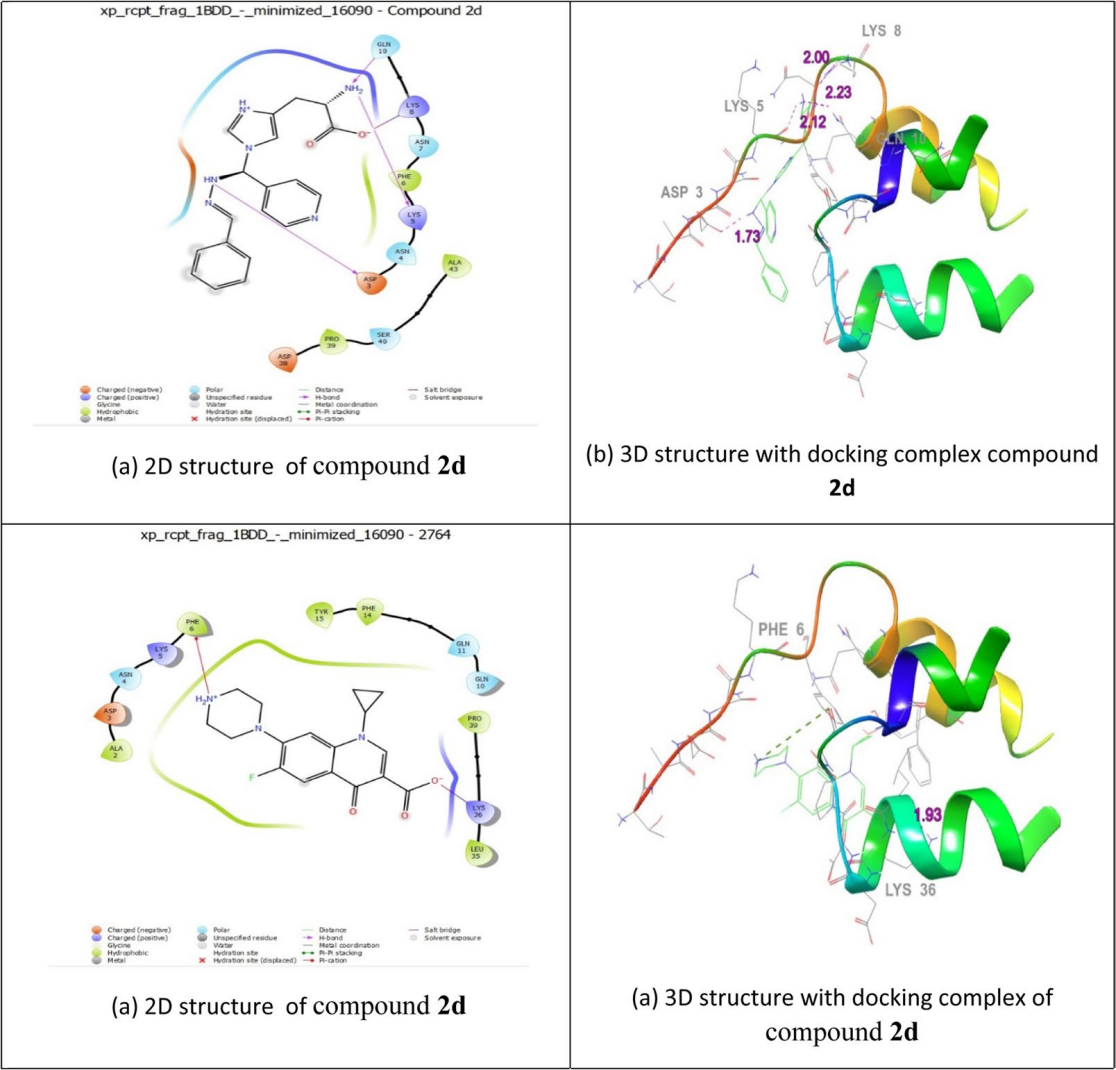
The molecular docking studies of compound **2d** and Ciprofloxacin binding with 1BDD protein

**Fig. 4:**
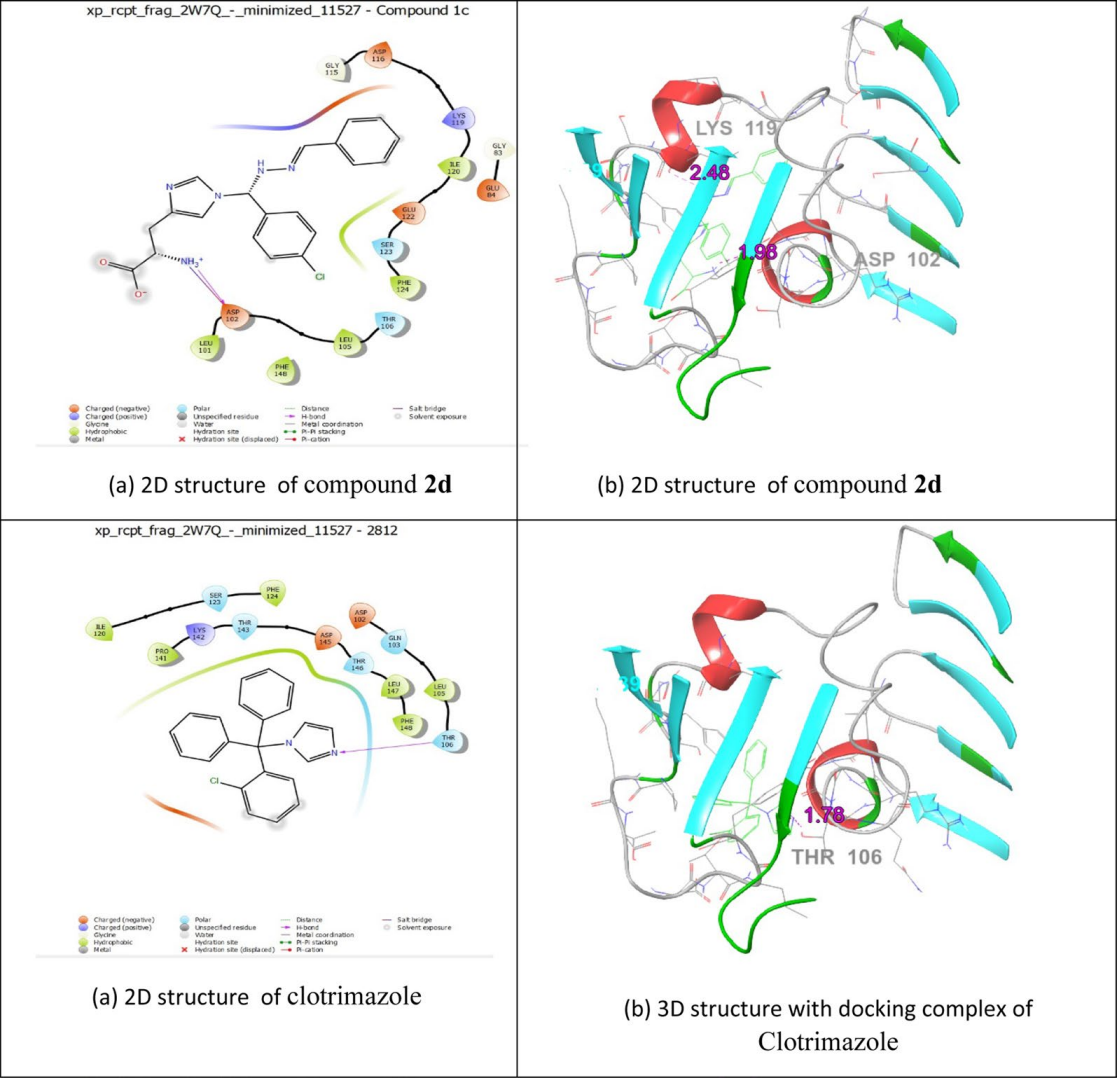
The molecular docking studies of compound **1c** and **Clotrimazole** binding with 1AI9 protein

**Fig. 5:**
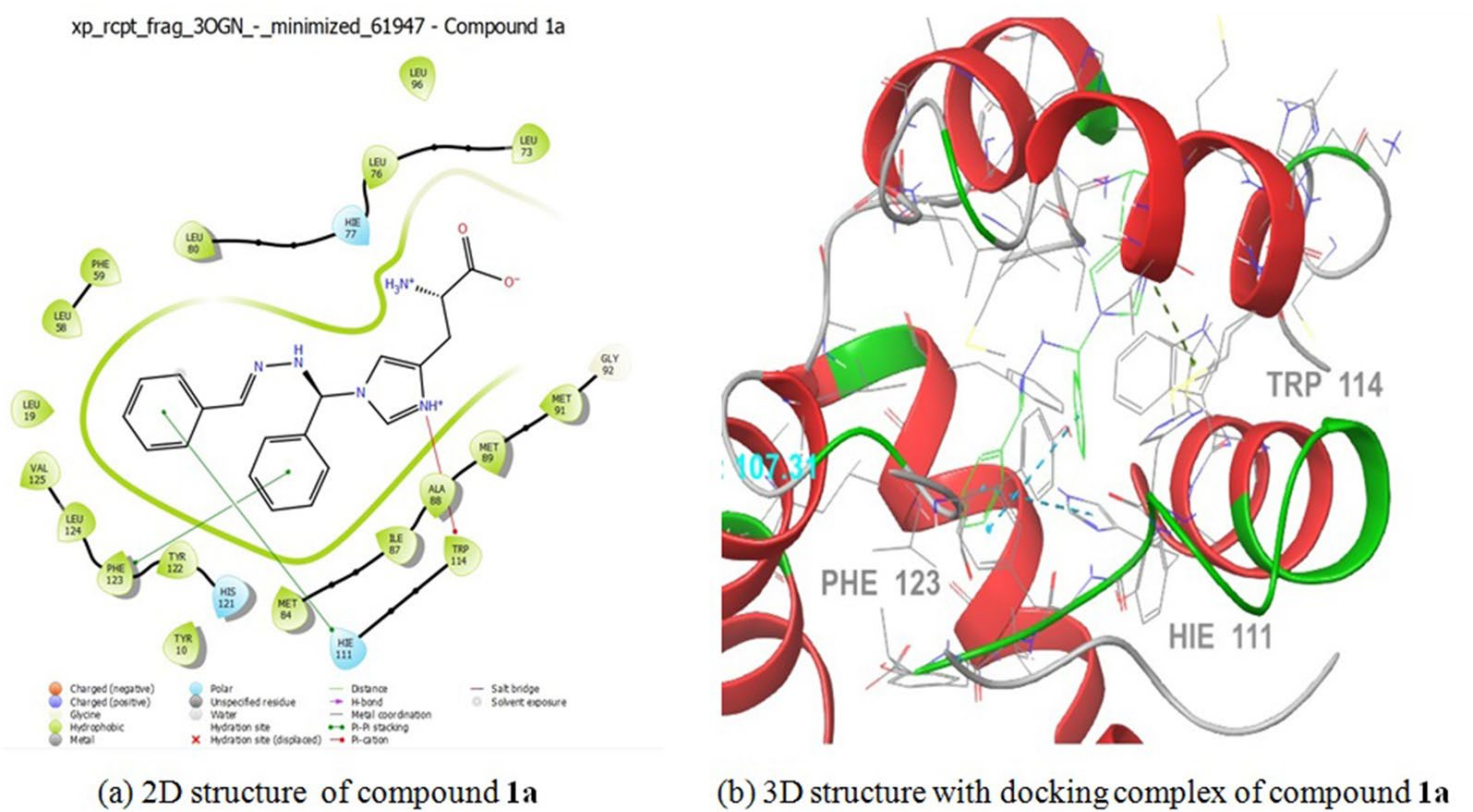
The molecular docking studies of compound **1a** binding with 3OGN Protein

ChemDraw Ultra software used for draw the 3D structures of **2d, 1c,** and **1a** (Figs. 3, 4, 5). The standard settings to support the Vina docking program were used for all other parameters that are not listed in this document. The substance with the lowest binding-affinity rating also had the highest score. All data were visually analysed using Discovery Studio 2019 software. Using the 1BDD, 1AI9, and 3OGN proteins in the Auto Dock Vina program, the docking abilities of the most effective synthetic compounds (**2d, 1c,** and **1a**) were investigated. In this case, the *S.aureus* protein binding score **2d** demonstrated a greater binding affinity for 1BDD (− 3.4 kcal/mol) and ciprofloxacin (− 4.4 kcal/mol) (Table 7). As a result, interacting residues were found in Asp 3, Lys 5, Lys 8, and Gln 10, with respect bond lengths of 1.73, 2.12, 2.00, and 2.23 compared with the standard ciprofloxacin found in Phe 6 and Lys 36, corresponding to bond lengths of 5.76, 1.93, respectively.

**Table 7:** Molecular Docking Interactions of** 2d, 1c, and 1a**

Protein Id	Compound Name	Dock Score (kcal/mol)	Interacting Residuces	Bond Length
1BDD	2d	− 3.4	Asp 3, Lys 5, Lys 8, Gln 10	1.73, 2.12, 2.00, 2.23
	Ciprofloxacin	− 4.4	Phe 6, Lys 36	5.76, 1.93
1AI9	1c	− 6.0	lle 19, Phe 36, lle 112, Ala 115	2.54, 4.81, 2.29, 1.94
	Clotrimazole	− 3.1	Thr 106	1.76
3OGN	1a	− 6.1	His 111, Trp 114, Phe 123	5.44, 5.29, 4.01

Antifungal activity of compound **1c** was establishes two hydrogen-bonding connections with receptor 1AI9. The docking score (− 6.0 kcal/mol) was compared with that of clotrimazole (− 3.1 kcal/mol) with interacting residues involving Ile 19, Phe 36, Ile 112, and Ala 115, with bond lengths of 2.54, 4.81, 2.29, and 1.94, compared with clotrimazole Thr 106 with band length 1.76, receptivity. Larvicidal activity **1a** (− 6.1 kcal/mol) interactions with the 3OGN protein and its receptors involved two hydrogen bonds. In this instance, the interactions involving residues His 111, Trp 114,Â and Phe 123, which had bond lengths of 5.44, 5.29, and 4.01, respectively, in the molecular docking interaction of 3OGN protein with permethrin, as detailed in our previous study [22]. Overall, the findings revealed that compounds **2d, 1c,** and **1a** had more antibacterial, antifungal, and larvicidal activities than the reference standards.

### HOMO–LUMO analysis

**Fig. 6:**
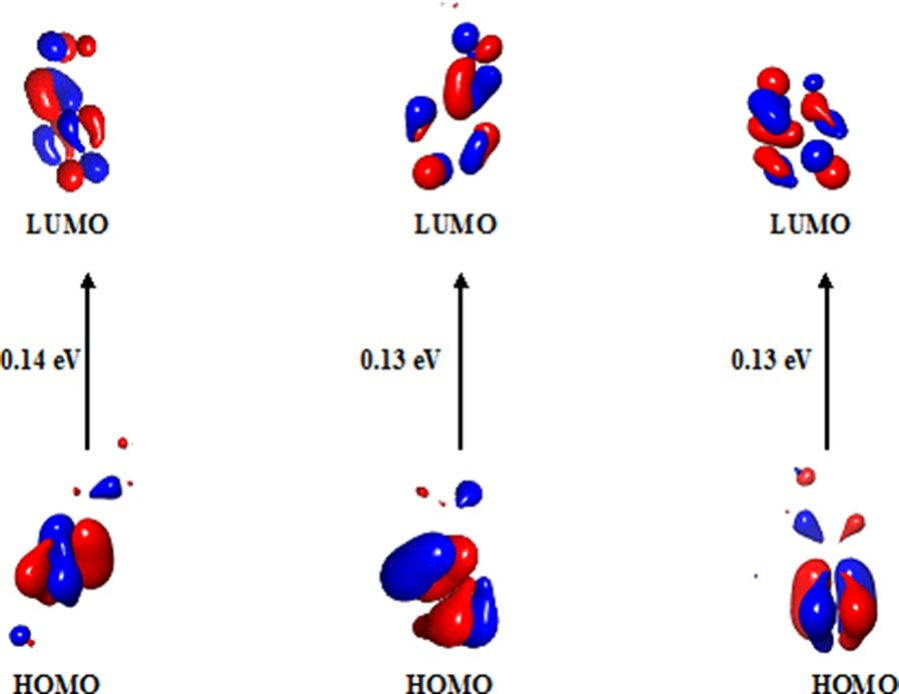
HOMO–LUMO energy diagram of** 2c, 2a, 1c**

The most crucial components of the HOMO–LUMO analysis are the electrical and chemical reactions of **2c, 2a,** and **1c**. "Donate an electron" and "receive an electron”, respectively, are the definitions of the acronyms HOMO and LUMO. As mentioned earlier, the forces behind the compounds are depicted in (Fig. 6) as the HOMO and LUMO energies, estimated using the DFT approach combined with the B3LYP/631G Basic Set (d, p). Generally, a compound is soft when the HOMO and LUMO energy differences are minimal, and complex when they are high. The parameters regarded the terms "lowest unoccupied molecular orbital" and "highest occupied molecular orbital" as acute in limiting the chemical stability and reactivity of the compound (Fig. 6).

The HOMO and LUMO energies of the two molecules were measured according to Koopman’s theorem, as shown in Table 8. Additionally, the energy values of the HOMO and LUMO were used to define the parameters ∆E gap (LUMO–HOMO energy), electrophilicity index (ω), electronegativity (χ), nucleophilicity index (N), global hardness (η), electron affinity (A), and ionisation energy (I), global softness (s), chemical potential (μ). These variables were calculated based on the previously mentioned equations and were connected. Because HOMO orbitals tend to give away electrons and LUMO orbitals tend to take electrons, their energies are proportional to their respective electron affinities (A) and ionisation energies (Ip). A large ∆E gap indicates an excellent stability and low chemical reactivity. ∆E gap is a measure of chemical reactivity. The results showed that **2c** (∆E gap = 0.14 eV) is more chemically stable than **2a** and **1c** (∆E gap = 0.13 eV). Global hardness (η), chemical potential (μ),global softness (S), and are additional standards for chemical stability. Higher hardness and lower softness values indicated the stability of the compound, for example compounds **2c**, **2a**, and **1c** (μ = 0.14 eV, η = 0.07 eV, and S = 14.3 eV). Compounds **2c, 2a,** and **1c** have Mulliken electronegativity (x) and Electrophilicity index (ω) values of x = 0.14 eV and ω = 0.14, respectively.

**Table 8:** Frontier molecular orbital energy and reactivity characteristics for **2a, 1c** and **2c**

Property	**2a**	**1c**	**2c**
HOMO	− 0.2	− 0.2	− 0.2
LUMO	− 0.07	− 0.07	− 0.06
Energygap ∆E (LUMO–HOMO)	0.13	0.13	0.14
Ionization Energy (I = εHOMO = − HOMO)	0.2	0.2	0.2
Electron Affinity (A = εLUMO = − LUMO)	0.07	0.07	0.06
Global Hardness (η = (I− A)/2)	0.07	0.07	0.07
Global Softness (s = 1/η)	14.3	14.3	14.3
Chemical Potential(μ = − (I + A)/2)	− 0.14	− 0.14	− 0.14
Electronegative(χ = − μ)	0.14	0.14	0.14
Electrophilicity Index (ω = μ2/2η)	0.14	0.14	0.14
Nucleophilicity Index (N = 1/ω)	7.14	7.14	7.14

### Molecular electrostatic potential surface

**Fig. 7:**
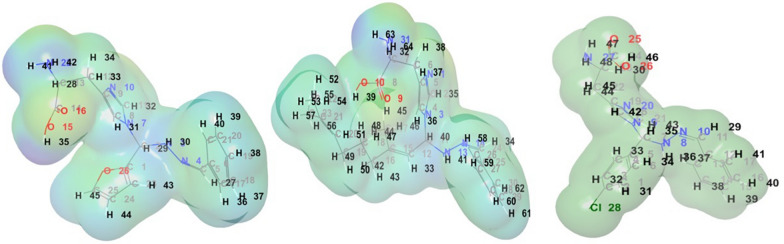
Electrostatic potential Map ** 2c, 2a** and ** 1c**

**Fig. 8:**
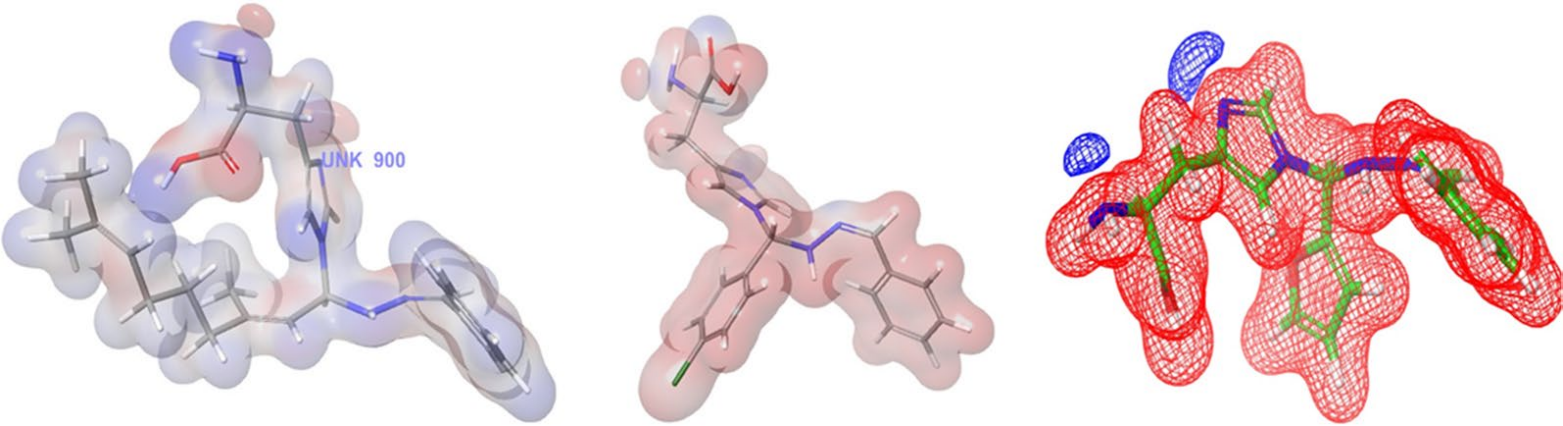
Electron density ** 2c, 2a** and ** 1c**

The potential surfaces provide information on the net electrostatic effects on the overall charge distribution of the molecule. A map of the molecular electron density surface is shown in (Figs. 7, 8), where the positive side of the nucleophilic atoms is coloured blue, and the positive side of the electrophilic atoms is green. The light-blue area indicates zero potential. The reactive regions in hydrogen bonds for nucleophilic and electrophilic attacks can be precisely identified with the help of MEP, which results from the charge distribution in space around a molecule.

## Conclusion

In this study, Cu(phen)Cl_2_ was used as a catalyst in the conversion process to create a series of Mannich-based imidazole derivatives, **1(a–f)** and **2(a–e)**. The Cu(phen)Cl_2_ catalyst was highly effective and yielded a higher yield than other Cu(II) catalysts. Compound **2d** (MIC: 0.25 μg/mL) was more active in *S. aureus* than ciprofloxacin (MIC: 0.5 μg/mL) with a molecular docking score of 1BDD protein (− 3.4 kcal/mol). The molecular docking score for compound **1c** for the 1AI9 protein was (− 6.0 kcal/mol) compared to clotrimazole's (− 3.1 kcal/mol), compound **1c** more effective in *C. albicans* (MIC = 0.25 g/mL). The molecular docking score of (− 6.1 kcal/mol) for the 3OGN protein of compound **1a**, larvicidal investigations showed that compound **1a** (LD_50_ = 34.9 g/mL) was significantly more effective than permethrin. Compounds **1a, 2d,** and **1c** can be considered to be the most potential compounds with larvicidal, antibacterial, and antifungal activities.

### Supplementary Information

**Additional file 1. **Figure S1 – S24: 1H NMR, 13C NMR, FTIR, and Mass spectrum of compounds (1a-1f) Figure S25- S42: 1H NMR, 13C NMR, FTIR, and Mass spectrum of compounds (2a-2e) Figure S43- S48: 13C spectra analysis labeled compounds (1a-1f) Figure S49-S53: 13C spectra analysis labeled compounds (2a-2e) Table S1-S6: 1H spectra analysis tabulation of compounds (1a-1f) Table S7-S11: 1H spectra analysis tabulation of compounds (2a-2e).

## Data Availability

The data and materials used in this study are entirely transparent.

## Abbreviations

DFT: Density functional theory
RNA: Ribonucleic acid
TLC: Thin-layer chromatography
