# Supplementary material for: Cu (II)-catalyzed: synthesis of imidazole derivatives and evaluating their larvicidal, antimicrobial activities with DFT and molecular docking studies
Source: BMC Chem. 2023 Nov 18;17(1):155. doi: 10.1186/s13065-023-01067-1 (PMC10657005; doi:10.1186/s13065-023-01067-1)
Supplement: Supplementary file 1 — Additional file 1. Figure S1 – S24: 1H NMR, 13C NMR, FTIR, and Mass spectrum of compounds (1a-1f) Figure S25- S42: 1H NMR, 13C NMR, FTIR, and Mass spectrum of compounds (2a-2e) Figure S43- S48: 13C spectra analysis labeled compounds (1a-1f) Figure S49-S53: 13C spectra analysis labeled compounds (2a-2e) Table S1-S6: 1H spectra analysis tabulation of compounds (1a-1f) Table S7-S11: 1H spectra analysis tabulation of compounds (2a-2e). [file 13065_2023_1067_MOESM1_ESM.pdf]

Cu (II)-Catalyzed: Synthesis of Imidazole Derivatives and Evaluating their Larvicidal, Antimicrobial activities with DFT and Molecular Docking Studies

Janani Mullaivendhan [a], Idhayadhulla Akbar [a]*, Mansour K. Gatasheh [b], Ashraf Atef Hatamleh [b], Anis Ahamed [b], Mohamed Hussain Syed Abuthakir [c]

Content

S.No.	Title	Page Number
1	^{1}H NMR and ^{13}C NMR, FTIR, EI-Mass Spectrum Of compounds (**1a**) – Figure S1-S4	2 - 3
2	^{1}H NMR and ^{13}C NMR, FTIR, EI-Mass Spectrum Of compounds (**1b**) – Figure S5-S8	4 - 5
3	^{1}H NMR and ^{13}C NMR, FTIR, EI-Mass Spectrum Of compounds (**1c**) – Figure S9-S12	6 - 7
4	^{1}H NMR and ^{13}C NMR, FTIR, EI-Mass Spectrum Of compounds (**1d**) – Figure S13-S16	8 - 9
5	^{1}H NMR and ^{13}C NMR, FTIR, EI-Mass Spectrum Of compounds (**1e**) – Figure S17-S20	10 - 11
6	^{1}H NMR and ^{13}C NMR, FTIR, EI-Mass Spectrum Of compounds (**1f**) – Figure S21-S24	12 - 13
7	^{1}H NMR and ^{13}C NMR, FTIR, EI-Mass Spectrum Of compounds (**2a**) – Figure S25-S28	14 - 15
8	^{1}H NMR and ^{13}C NMR, FTIR, EI-Mass Spectrum Of compounds (**2b**) – Figure S29-S32	16 - 17
9	^{1}H NMR and ^{13}C NMR, FTIR, EI-Mass Spectrum Of compounds (**2c**) – Figure S33-S36	18 - 19
10	^{1}H NMR and ^{13}C NMR, FTIR, EI-Mass Spectrum Of compounds (**2d**) – Figure S37-S40	20 - 21
11	^{1}H NMR and ^{13}C NMR, FTIR, EI-Mass Spectrum Of compounds (**2e**) – Figure S41-S42	22
12	^{1}H spectra analysis tabulation of compound **(1a-f)** and **(2a-e)** Table S1-S11	23-33
13	^{13}C spectra analysis labeled compound **(1a-f)** and **(2a-e)** Figure S43-S53	34-44

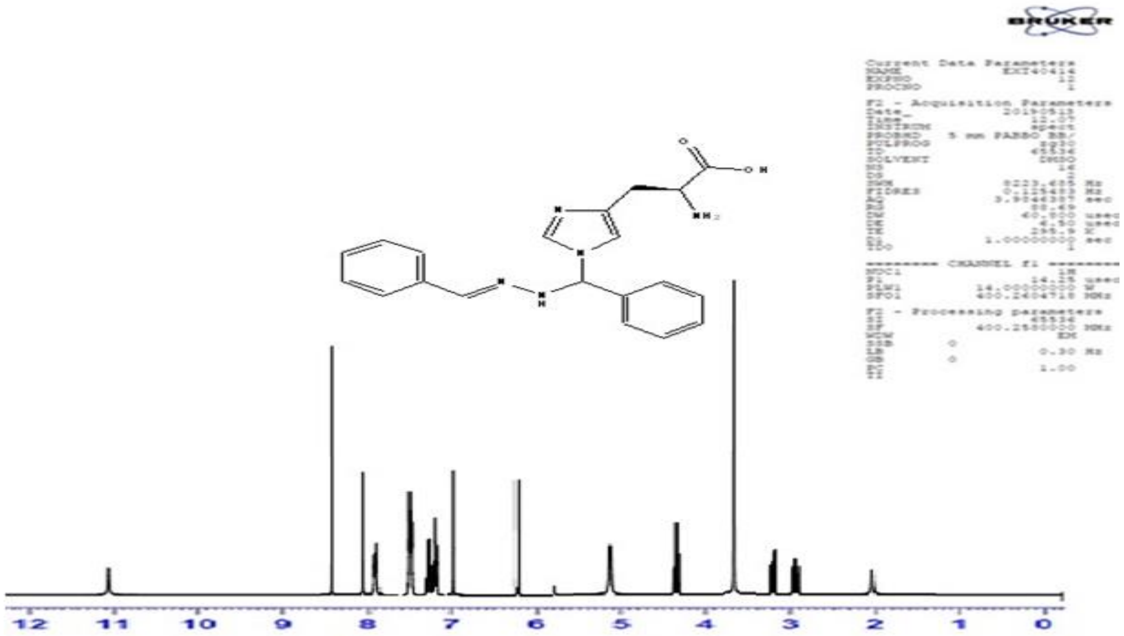

Figure S1 ^{1}H NMR spectrum of the compound **1a**

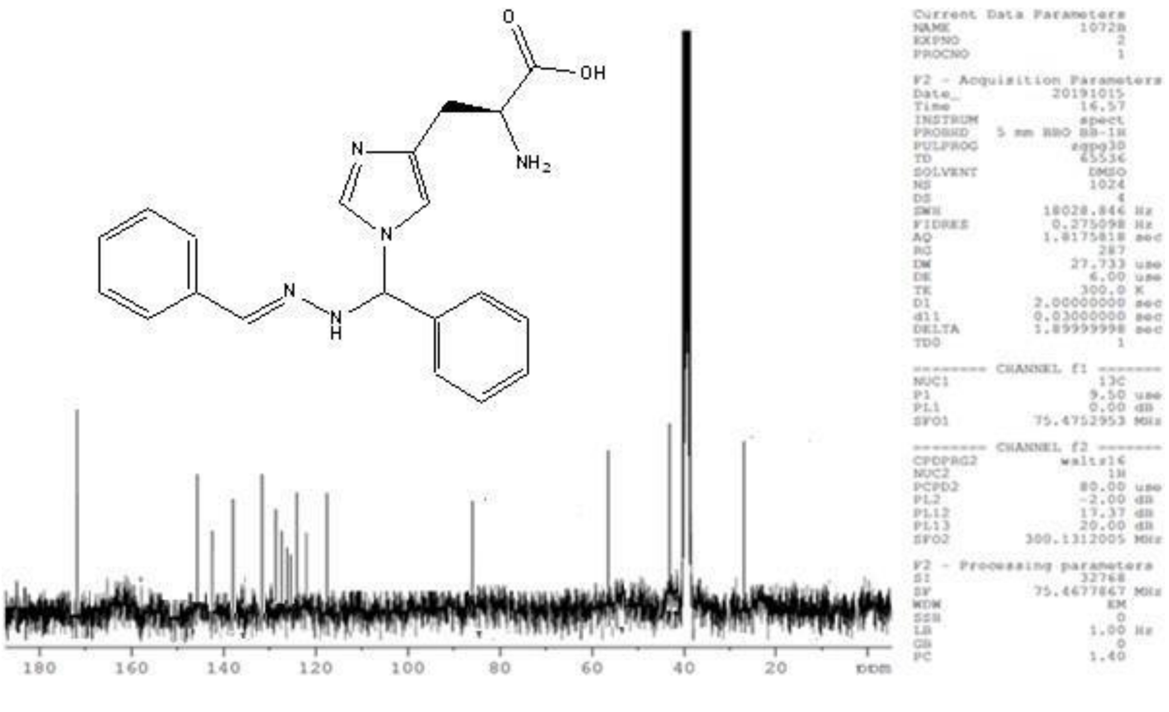

Figure S2 ^{13}C NMR spectrum of the compound **1a**

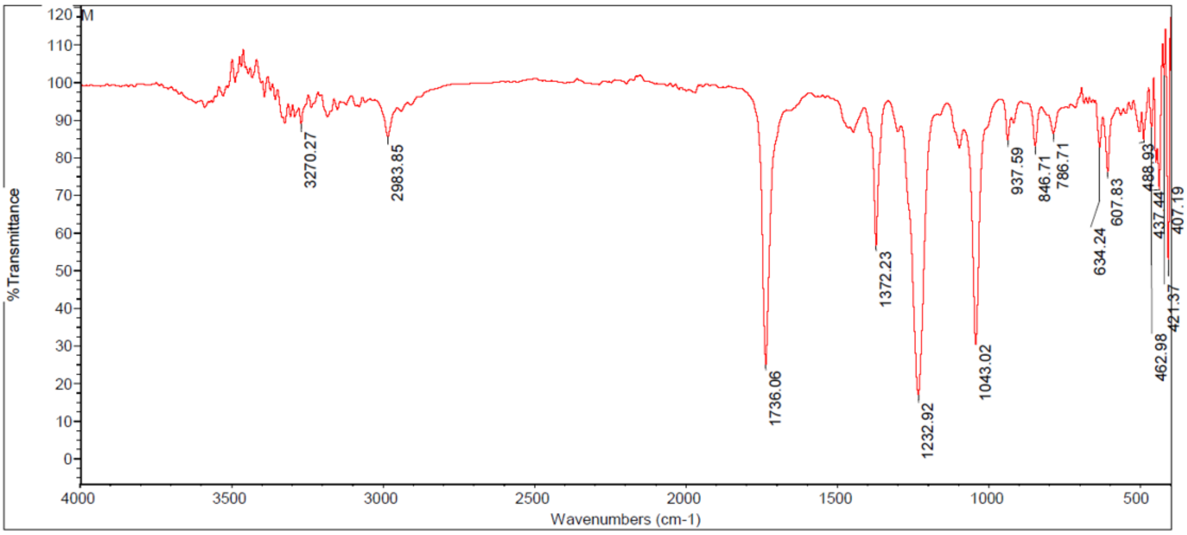

Figure S3 FTIR spectrum of the compound **1a**

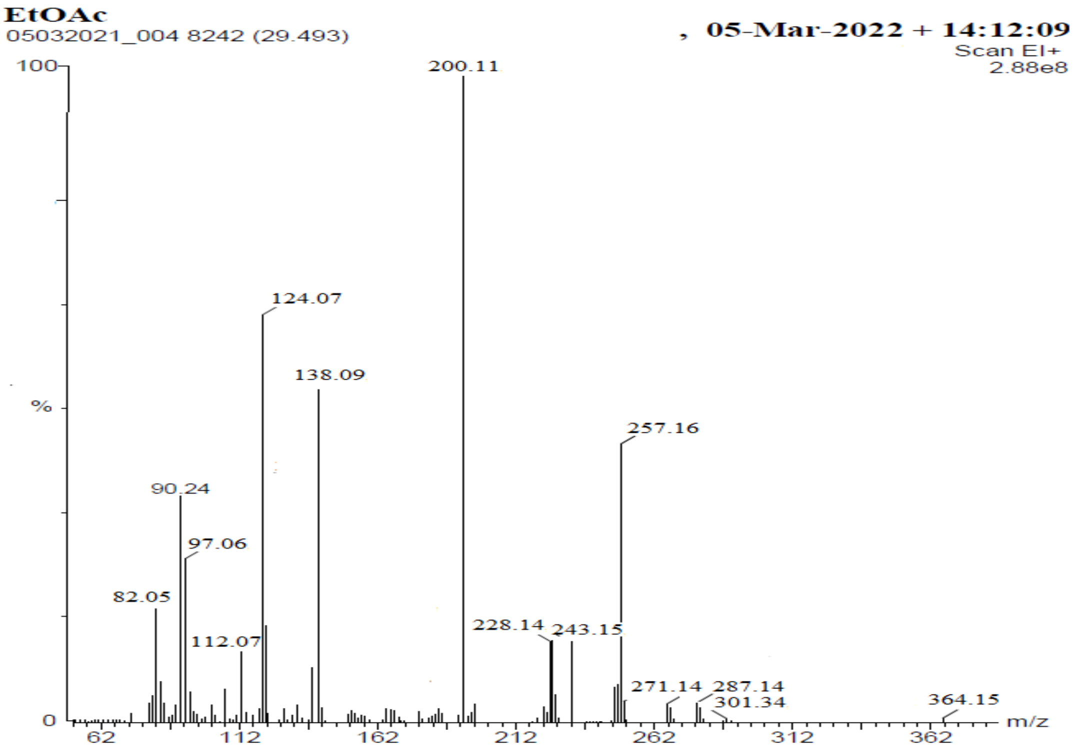

Figure S4 Mass spectrum of the compound **1a**

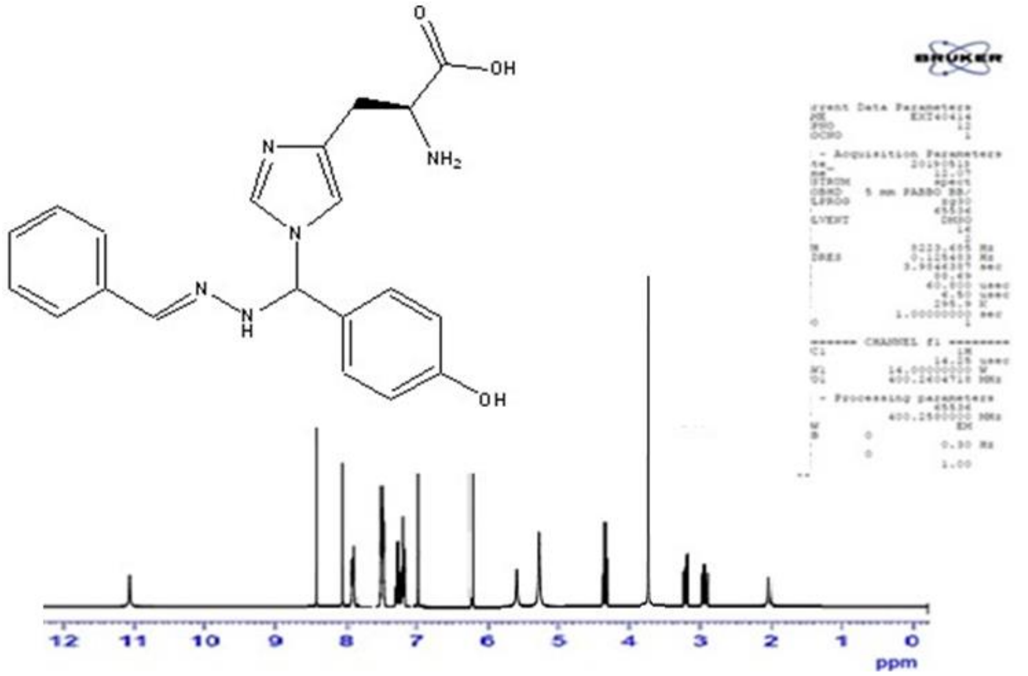

Figure S5 ^{1}H NMR spectrum of the compound **1b**

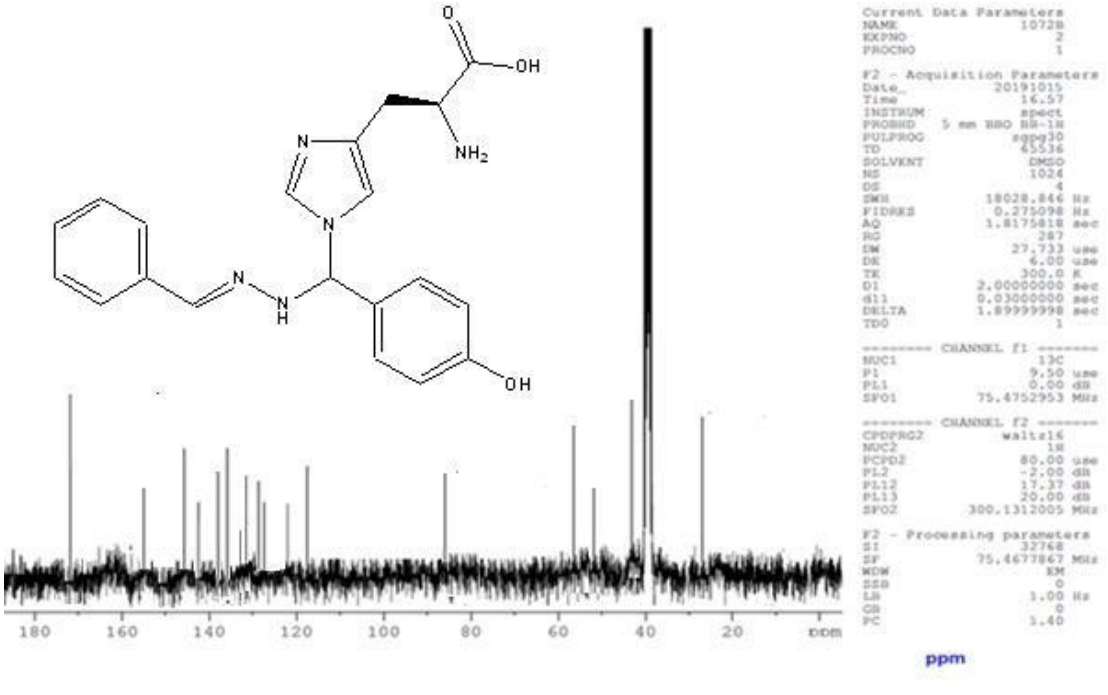

Figure S6 ^{13}C NMR spectrum of the compound **1b**

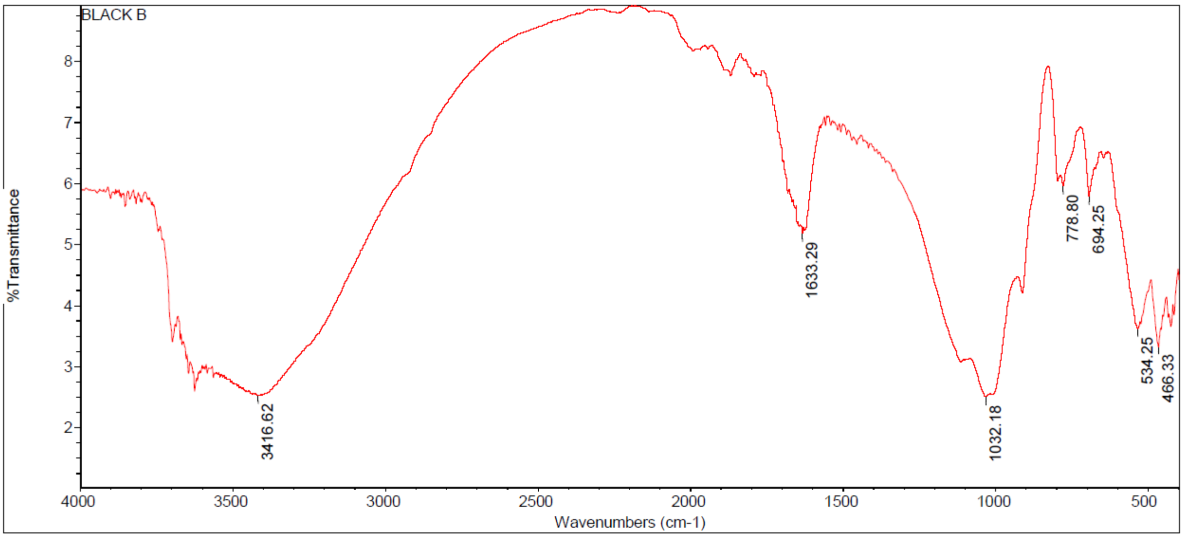

Figure S7 FTIR spectrum of the compound **1b**

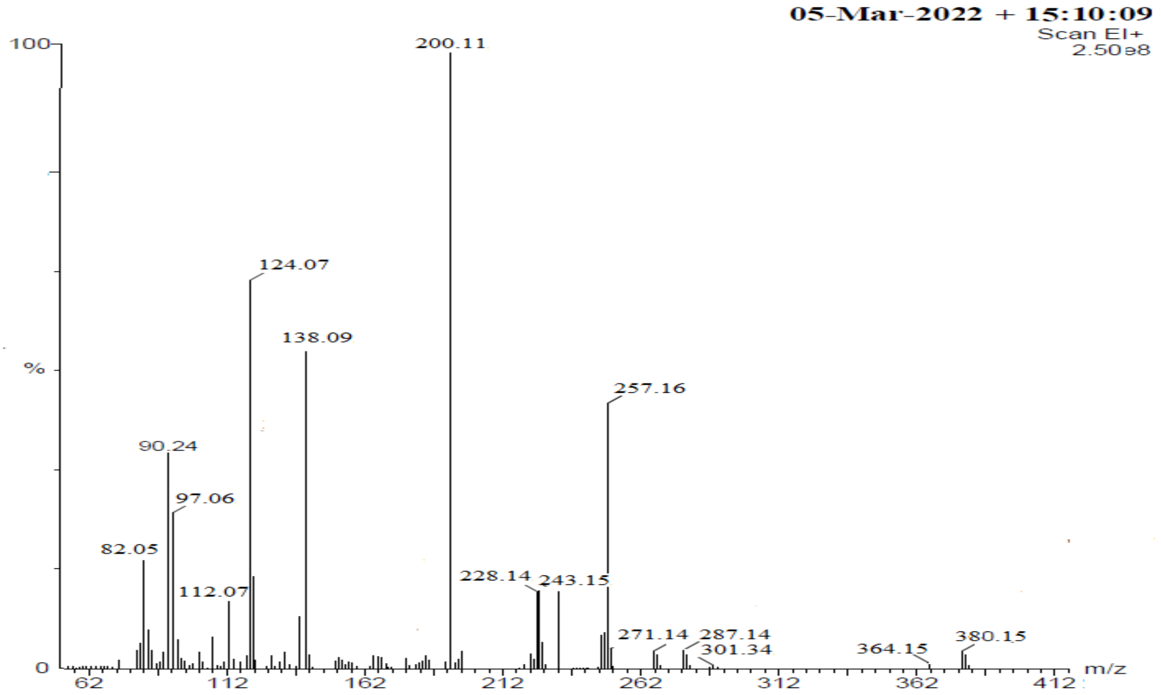

Figure S8 Mass spectrum of the compound **1b**

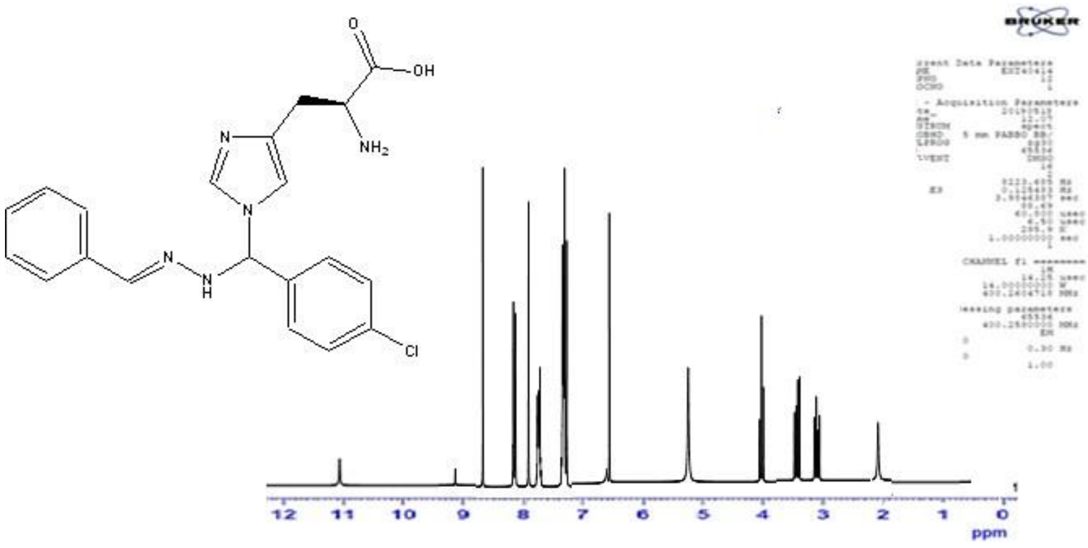

Figure S9 ^{1}H NMR spectrum of the compound **1c**

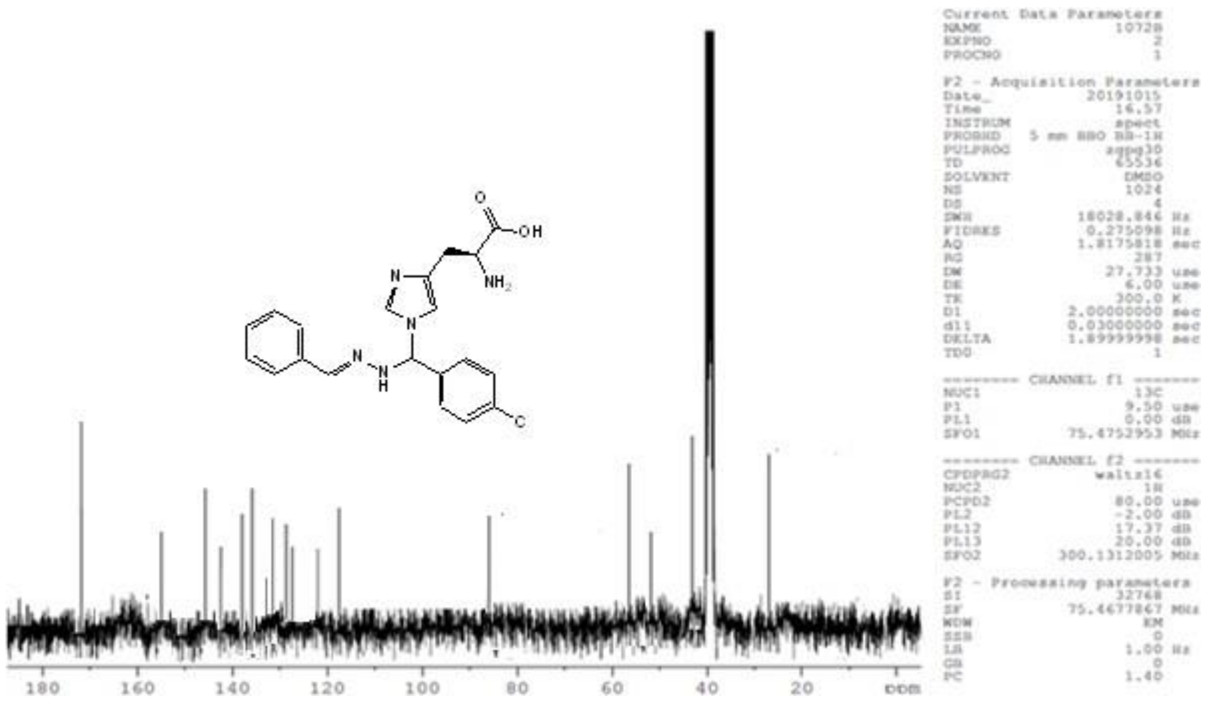

Figure S10 ^{13}C NMR spectrum of the compound **1c**

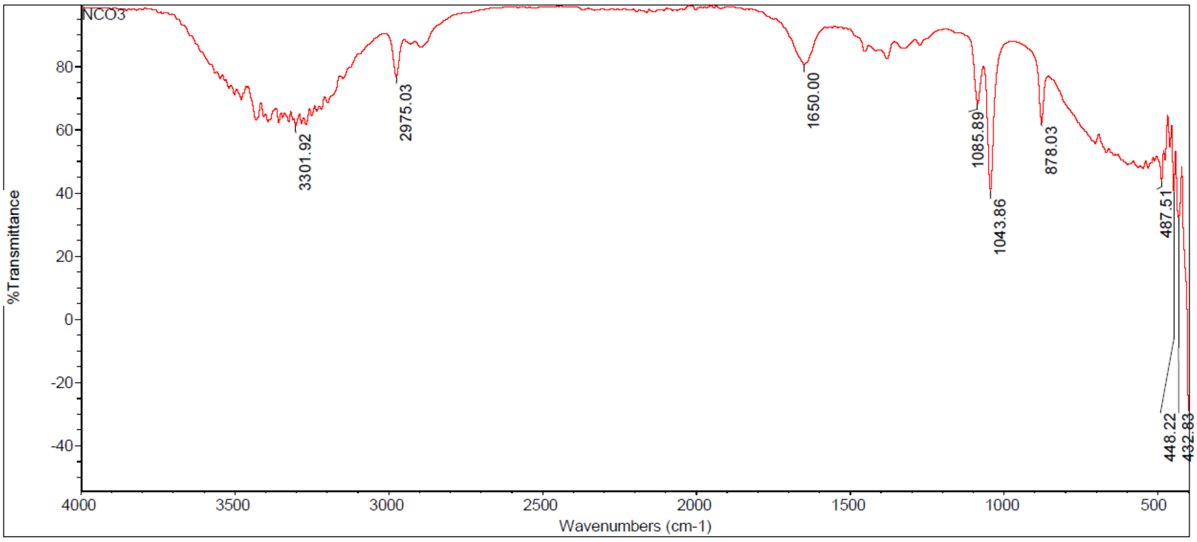

Figure S11 FTIR spectrum of the compound **1c**

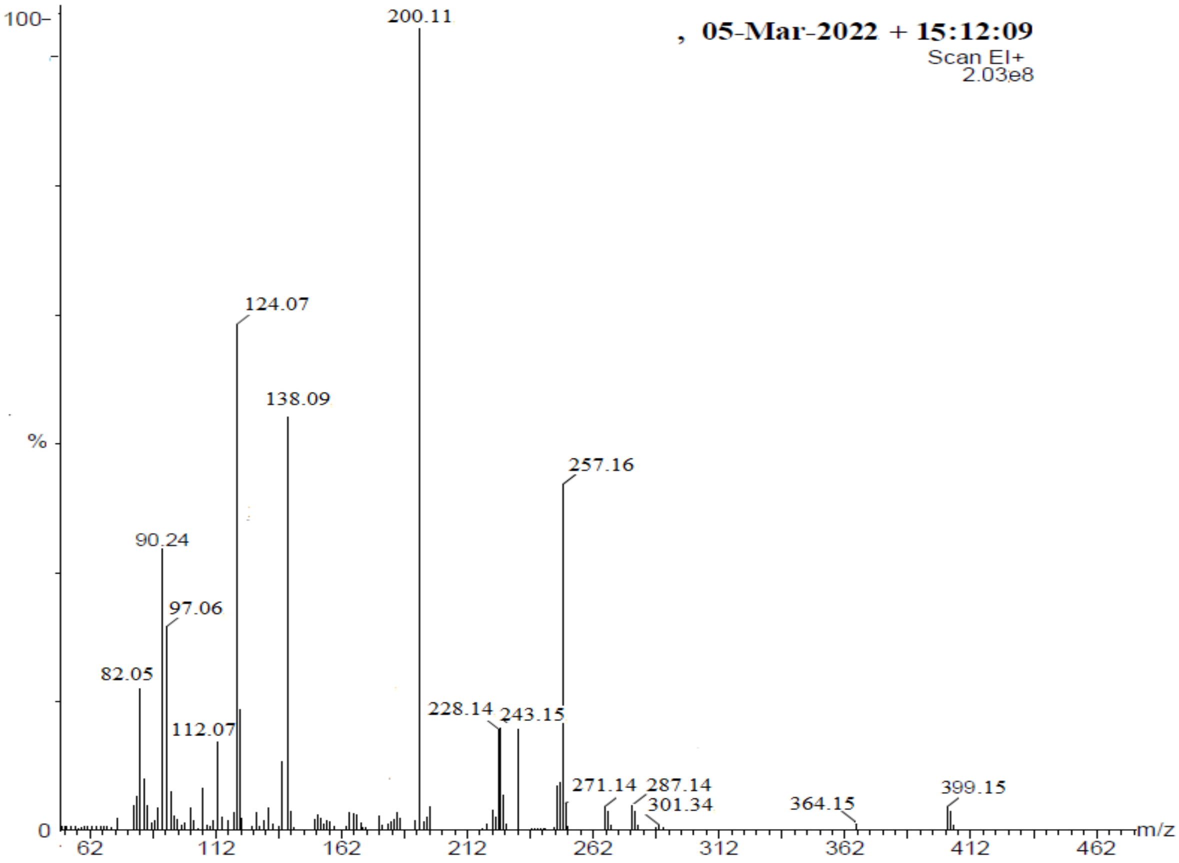

Figure S12 Mass spectrum of the compound **1c**

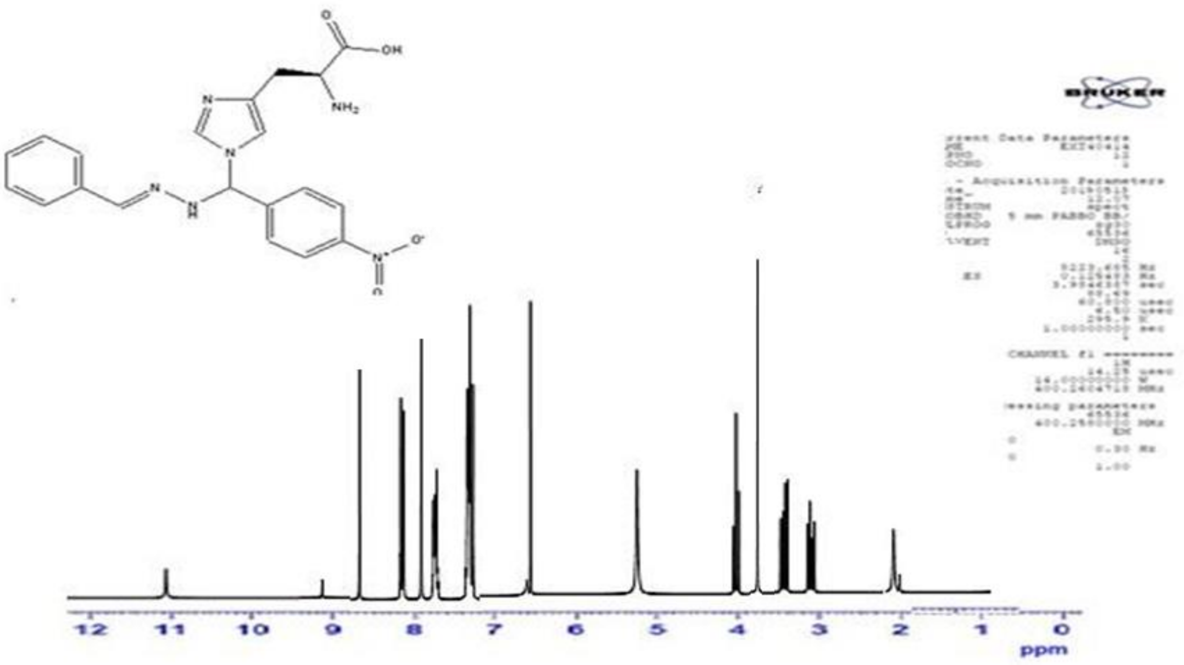

Figure S13 ^{1}H NMR spectrum of the compound **1d**

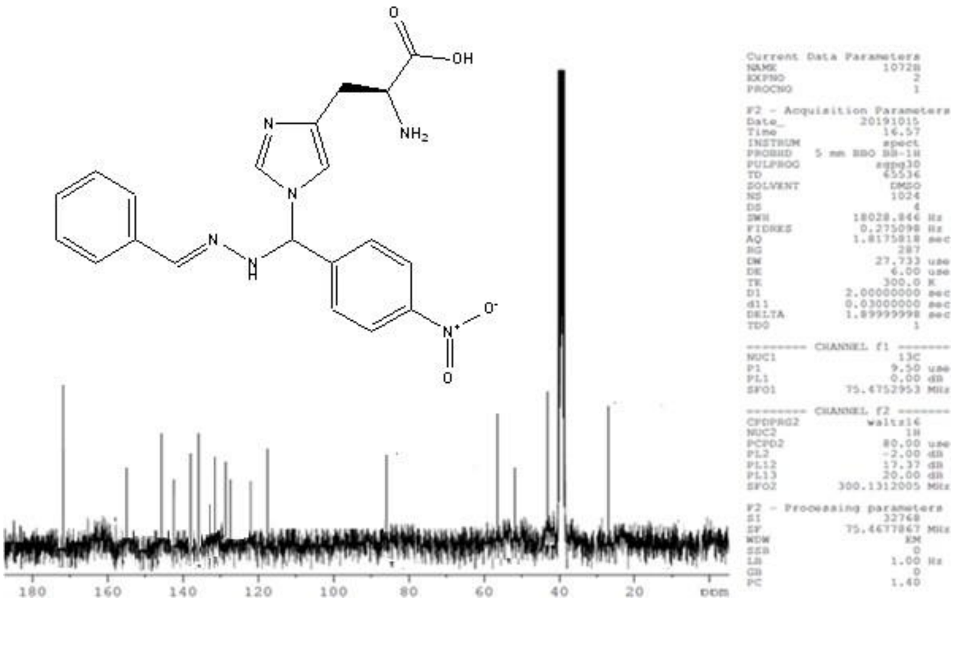

Figure S14 ^{13}C NMR spectrum of the compound **1d**

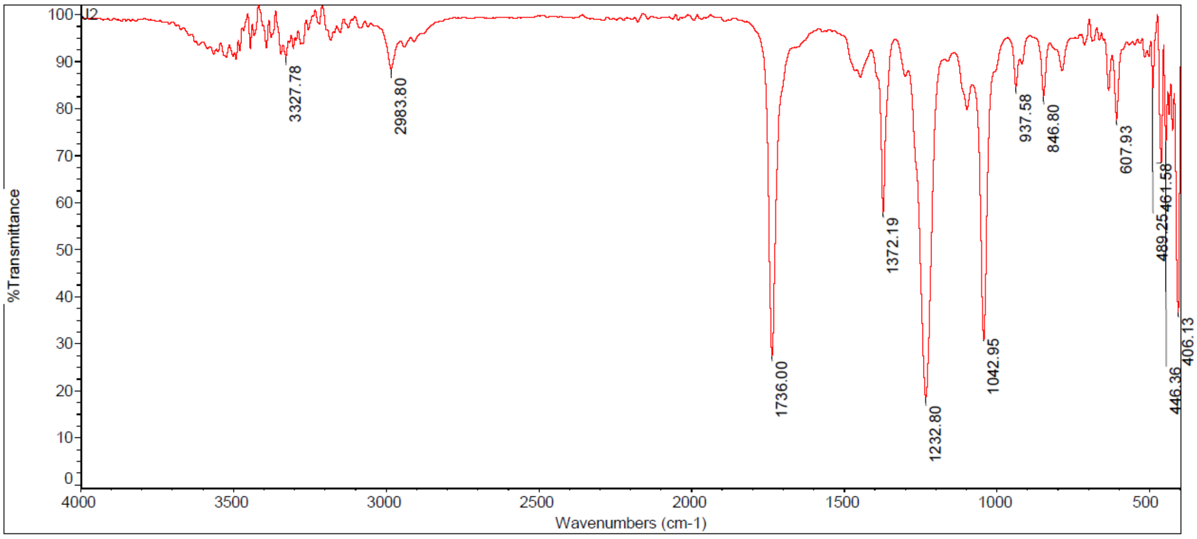

Figure S15 FTIR spectrum of the compound **1d**

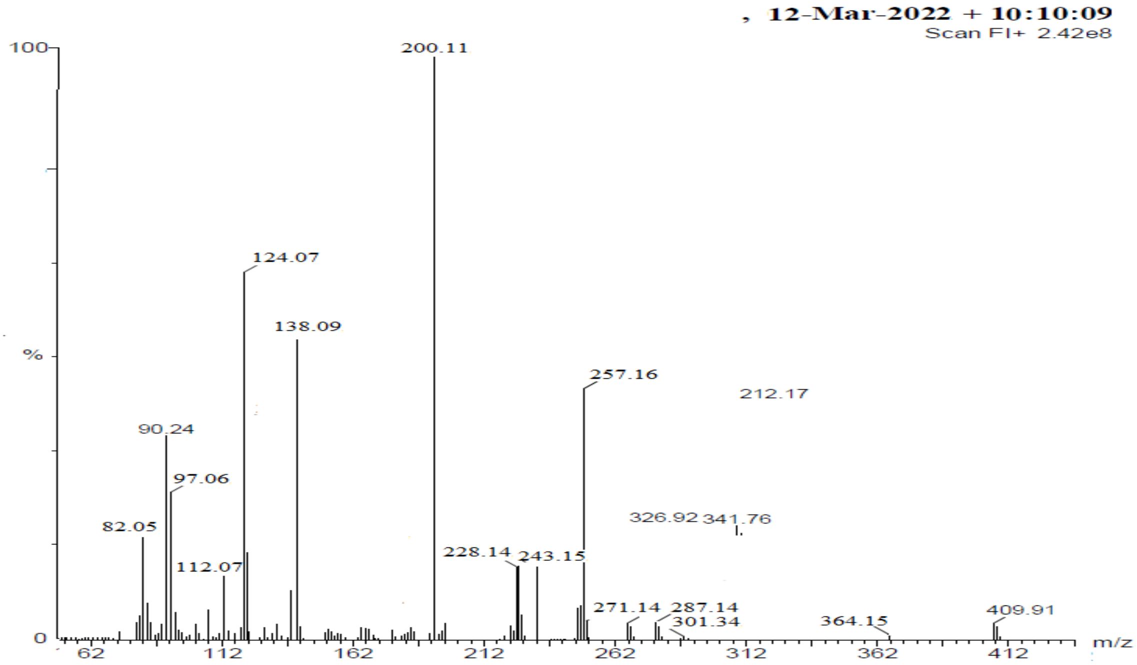

Figure S16 Mass spectrum of the compound **1d**

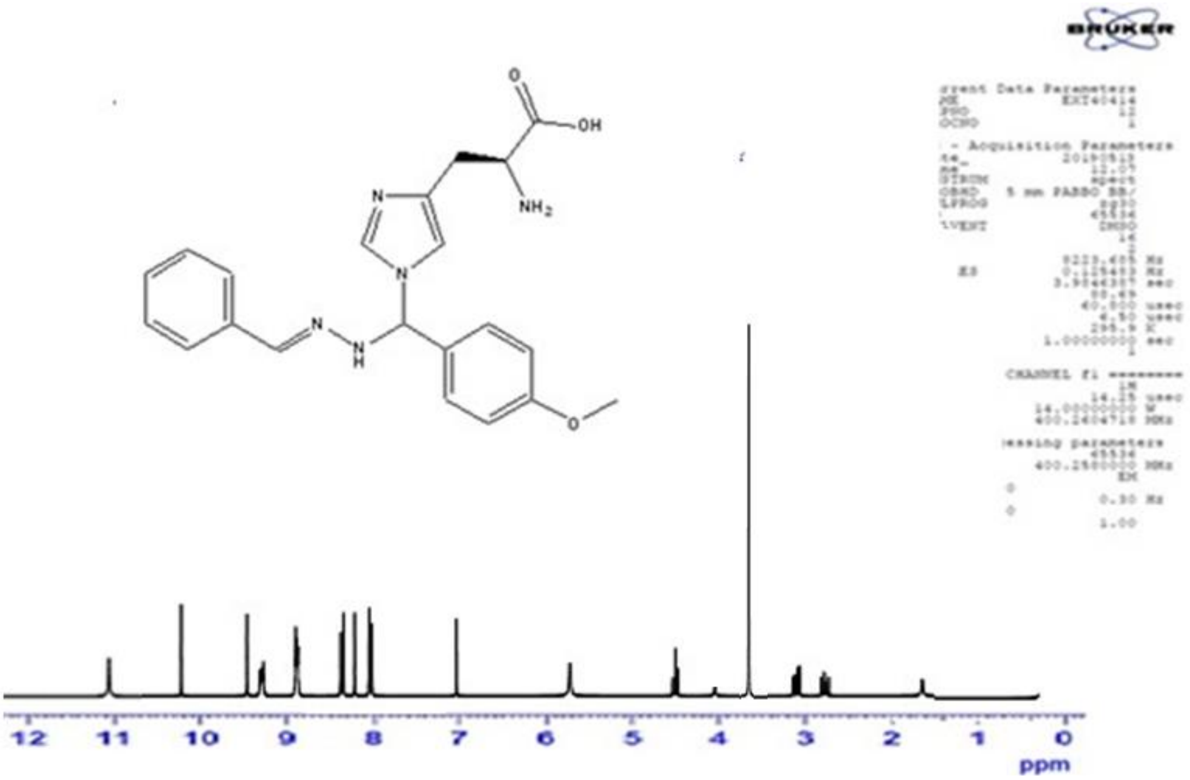

Figure S17 ^{1}H NMR spectrum of the compound **1e**

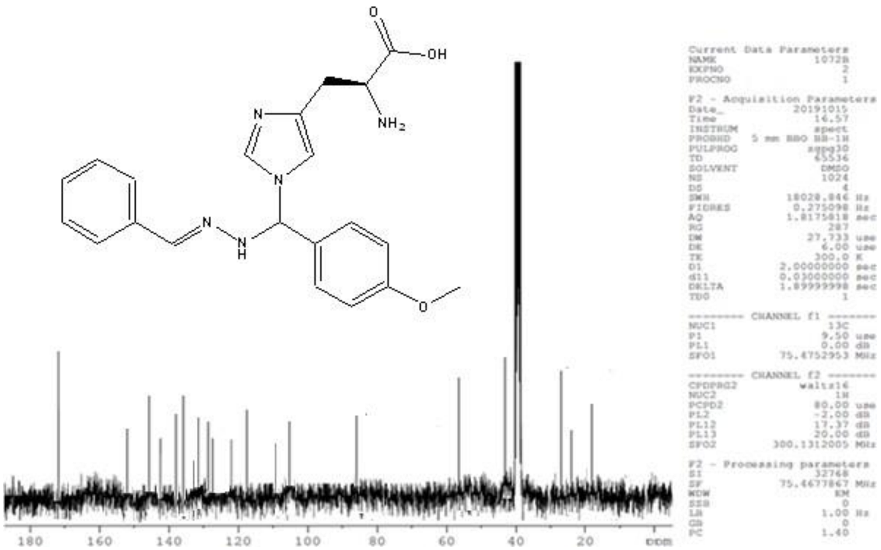

Figure S18 ^{13}C NMR spectrum of the compound **1e**

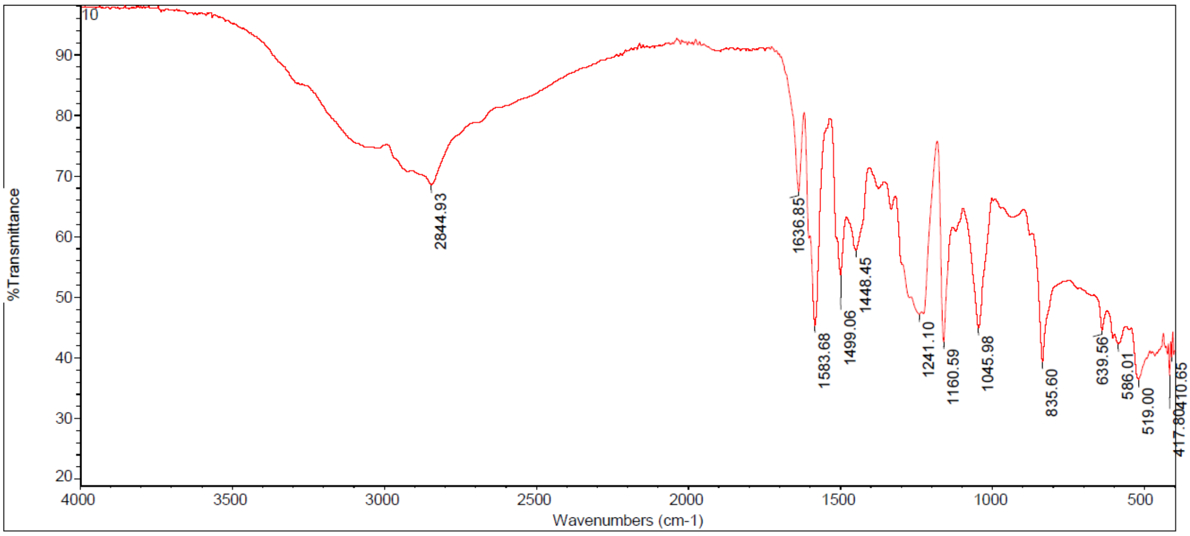

Figure S19 FTIR spectrum of the compound **1e**

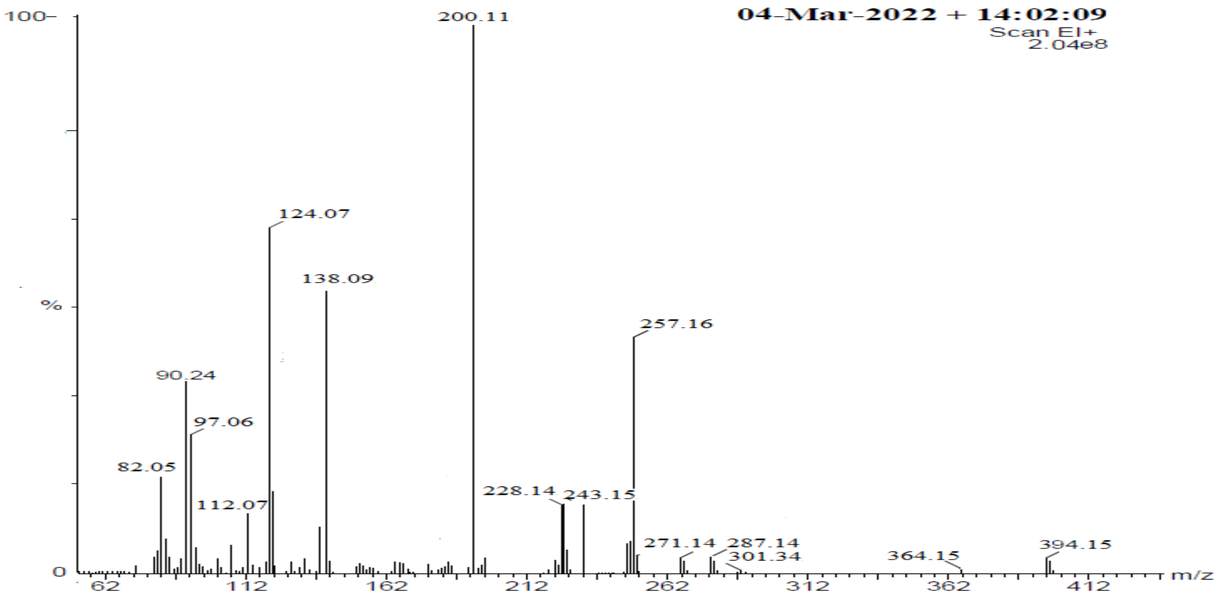

Figure S20 Mass spectrum of the compound **1e**

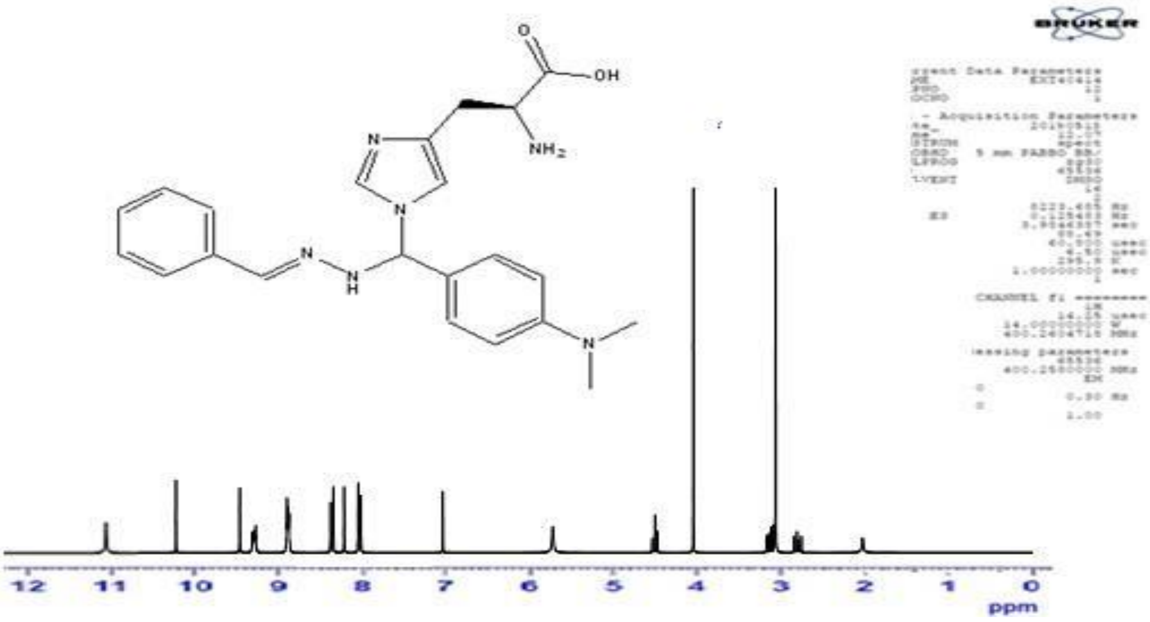

Figure S21 ^{1}H NMR spectrum of the compound **1f**

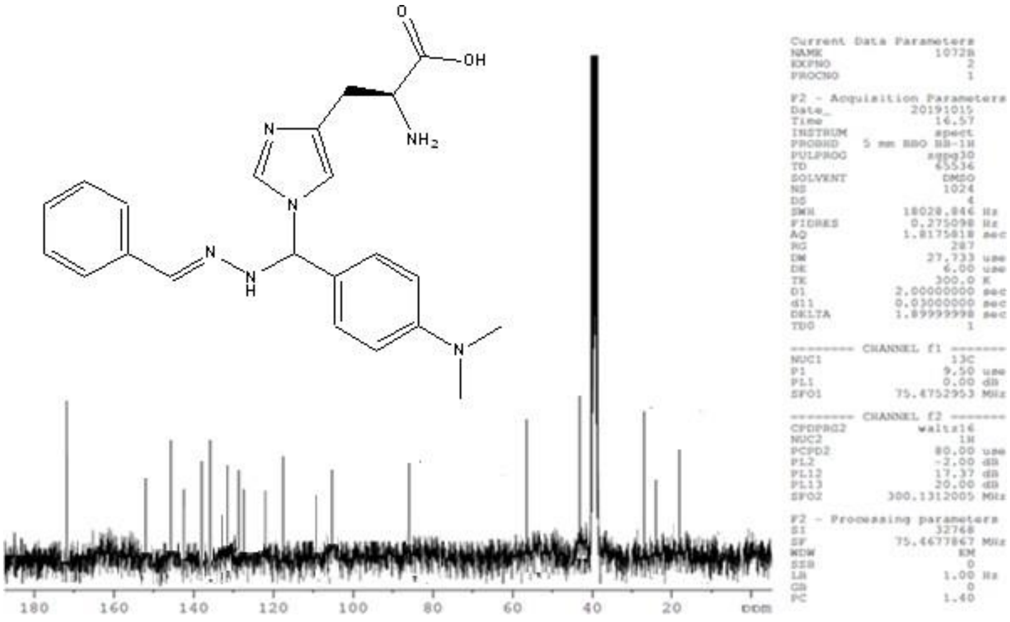

Figure S22 ^{13}C NMR spectrum of the compound **1f**

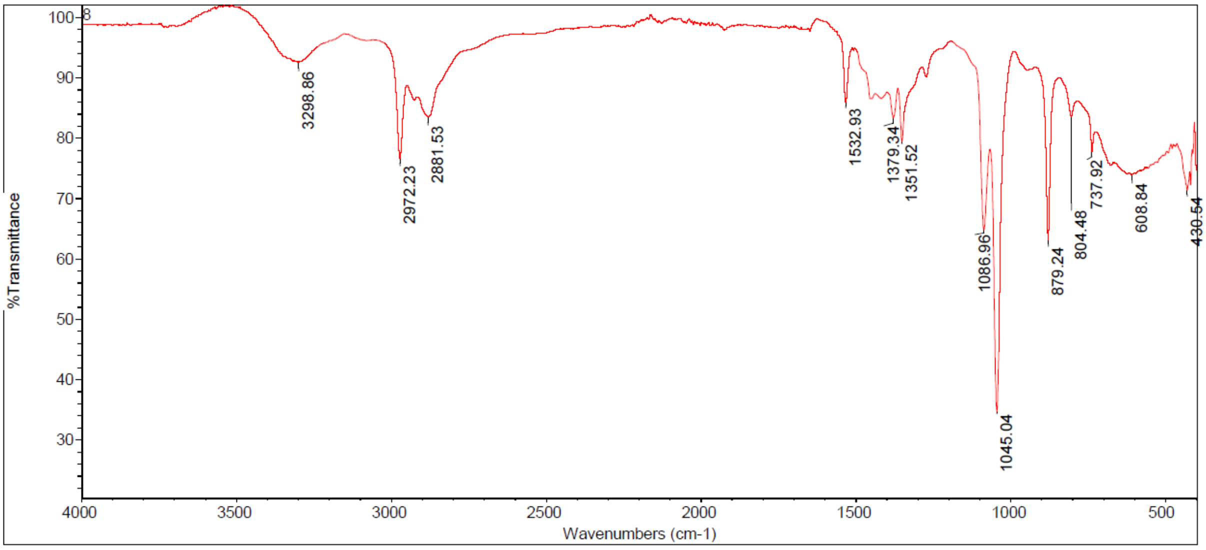

Figure S23 FTIR spectrum of the compound **1f**

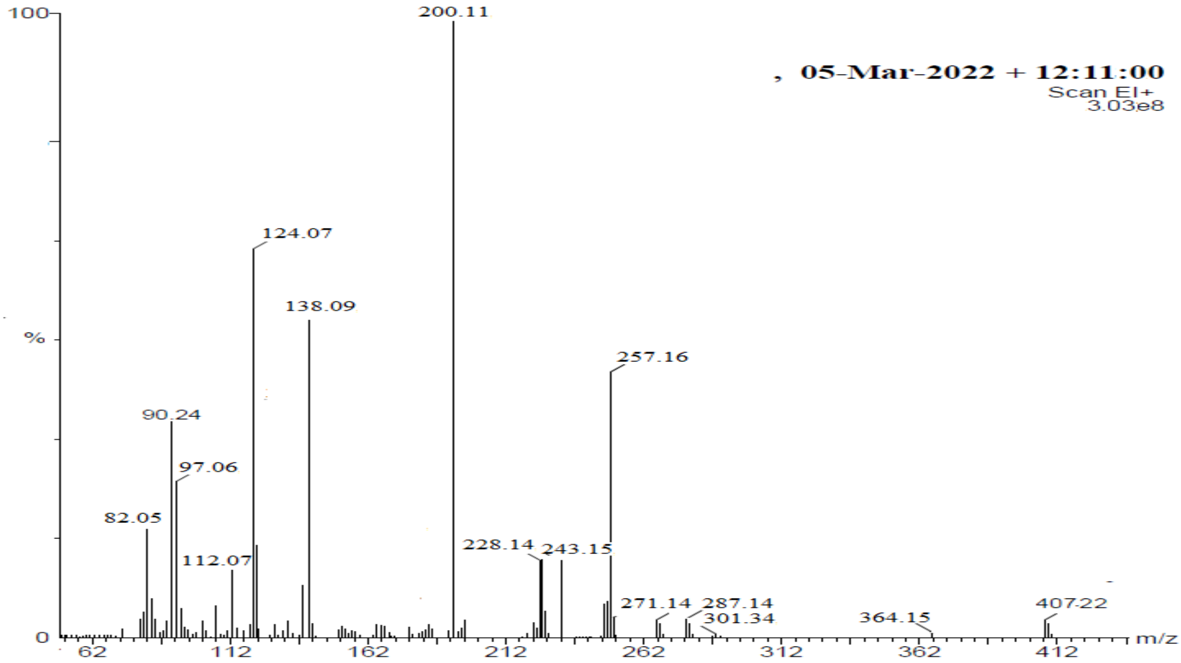

Figure S24 Mass spectrum of the compound **1f**

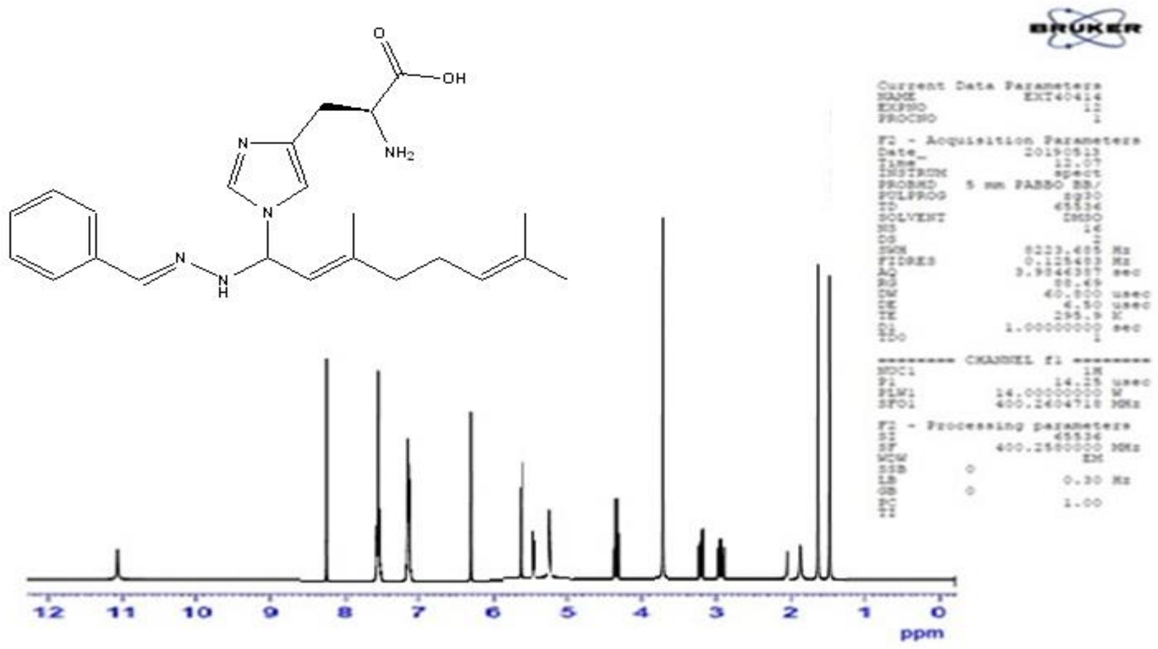

Figure S25 ^{1}H NMR spectrum of the compound **2a**

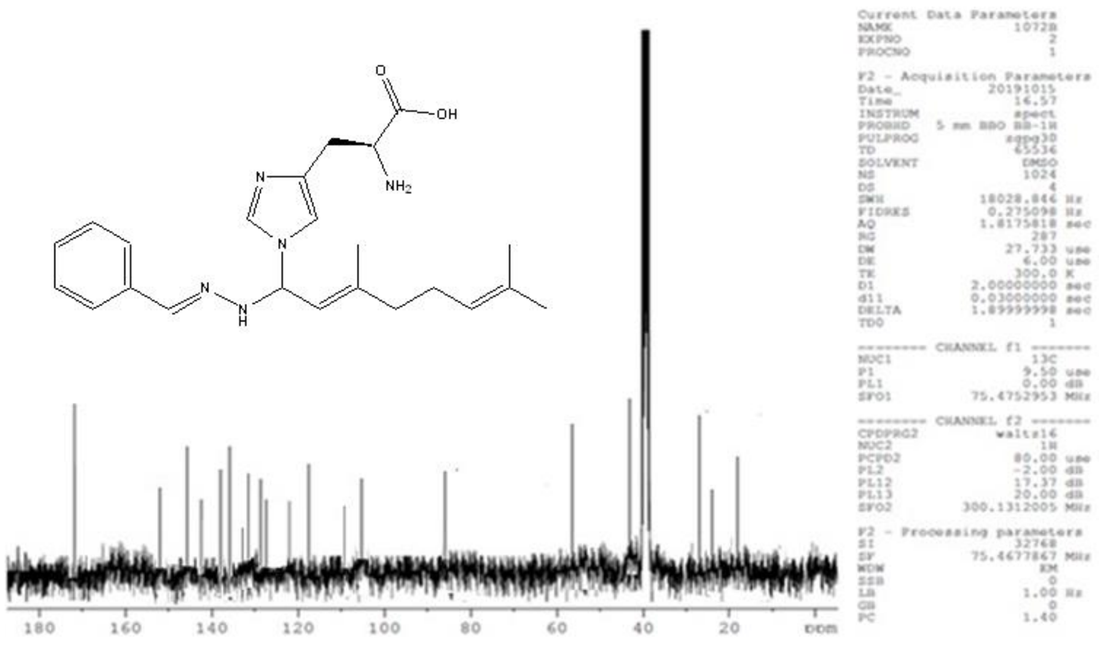

igure S26 ^{13}C NMR spectrum of the compound **2a**

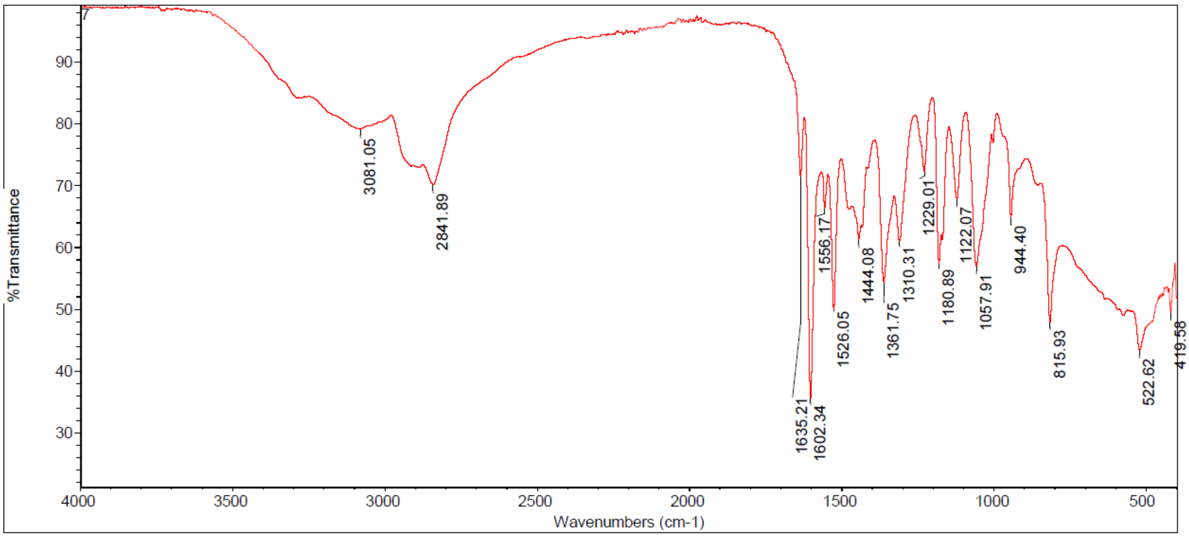

Figure S27 FTIR spectrum of the compound **2a**

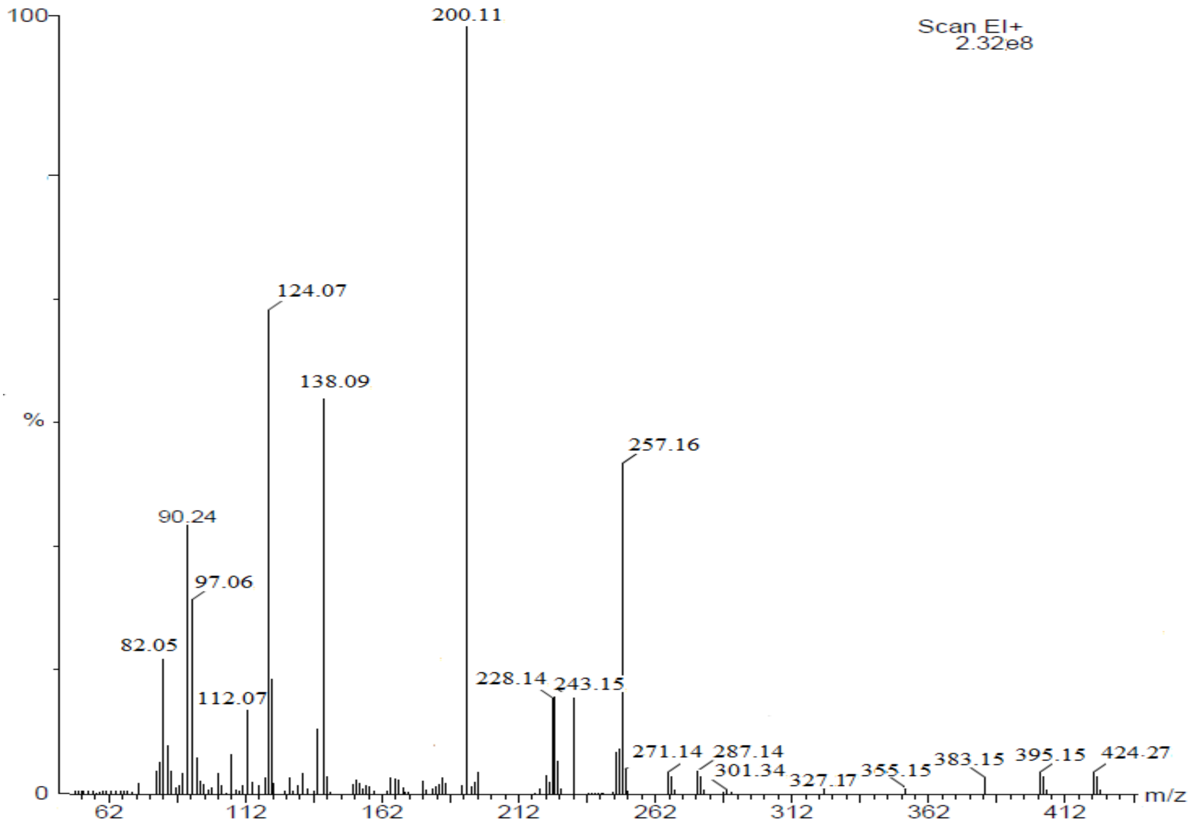

Figure S28 Mass spectrum of the compound **2a**

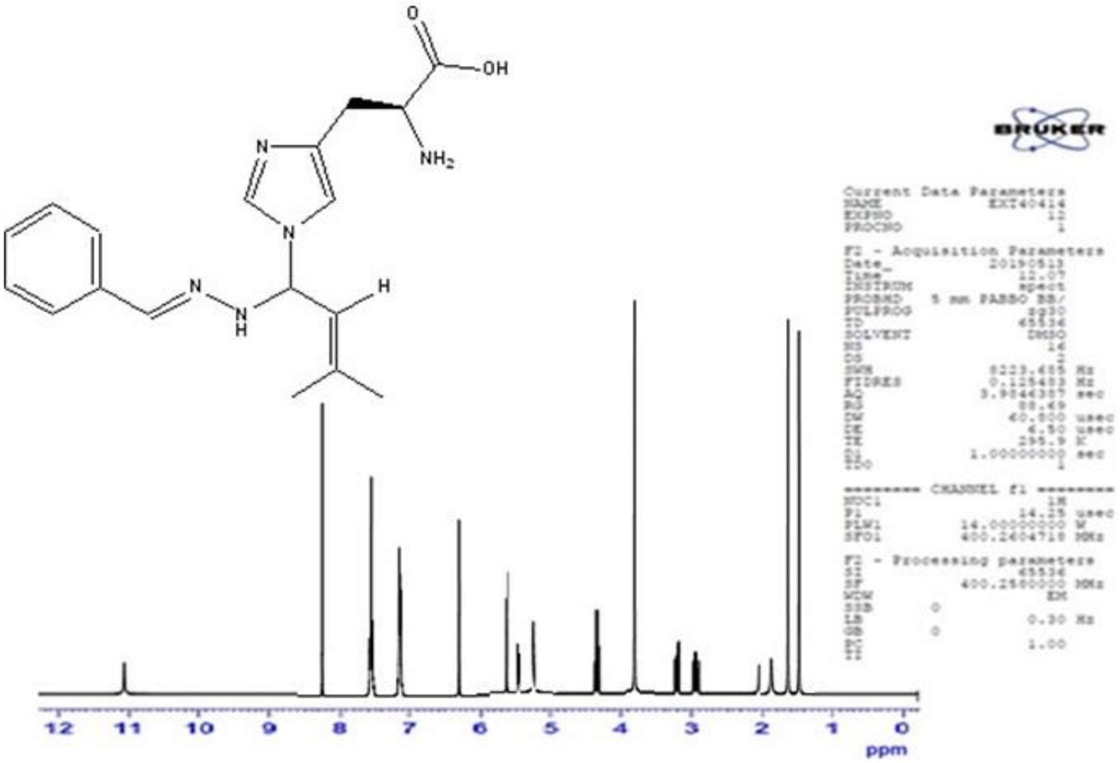

Figure S29 ^{1}H NMR spectrum of the compound **2b**

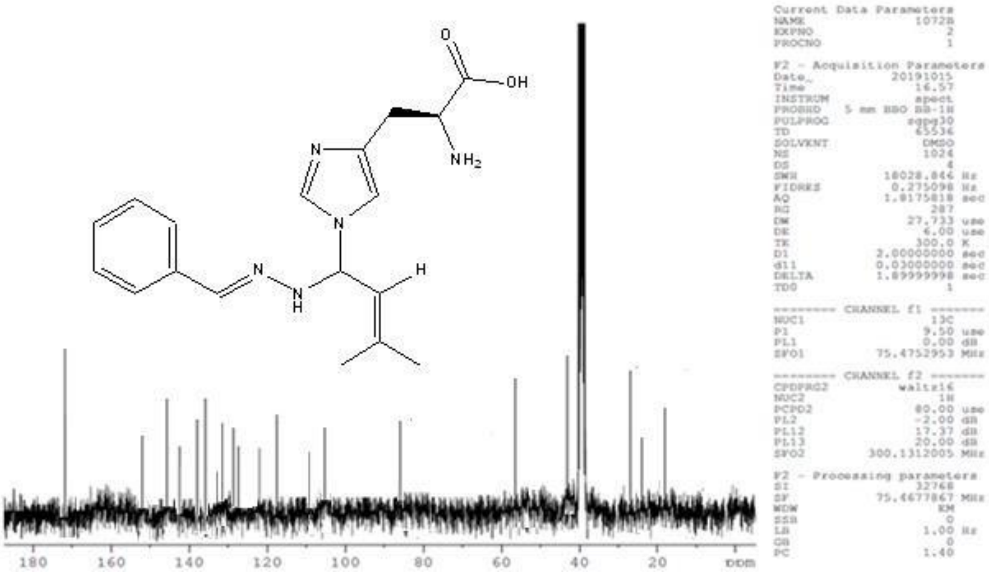

Figure S30 ^{13}C NMR spectrum of the compound **2b**

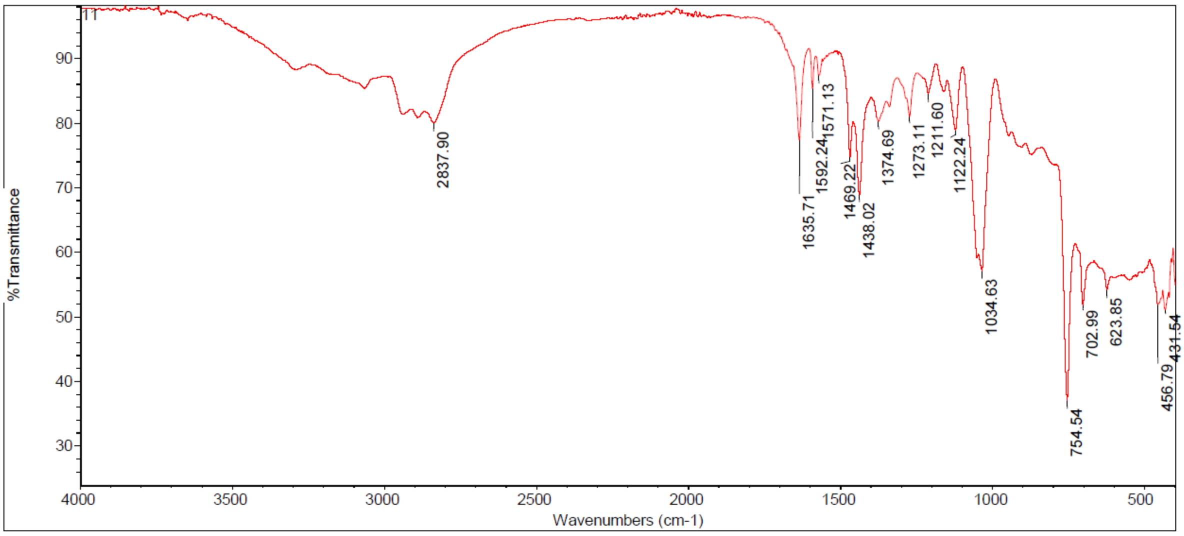

Figure S31 FTIR spectrum of the compound **2b**

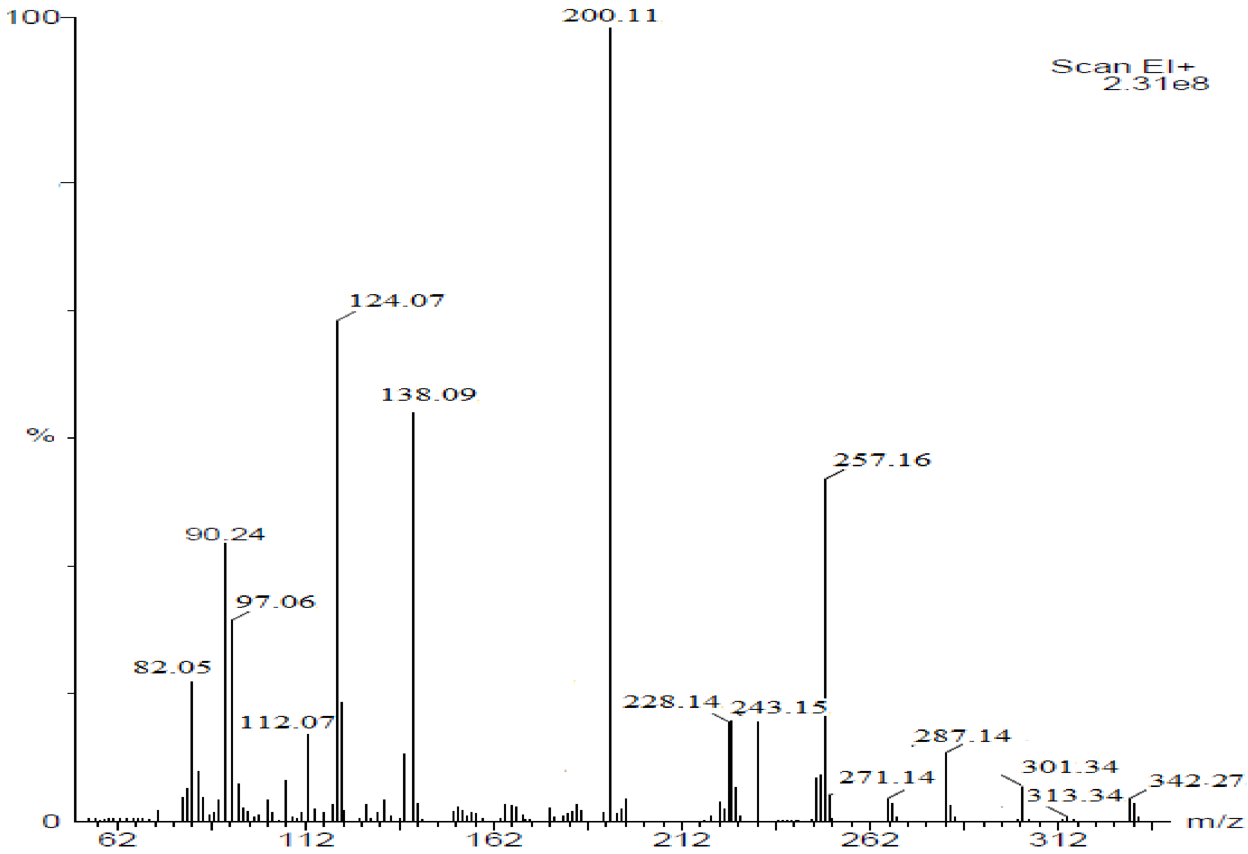

Figure S32 Mass spectrum of the compound **2b**

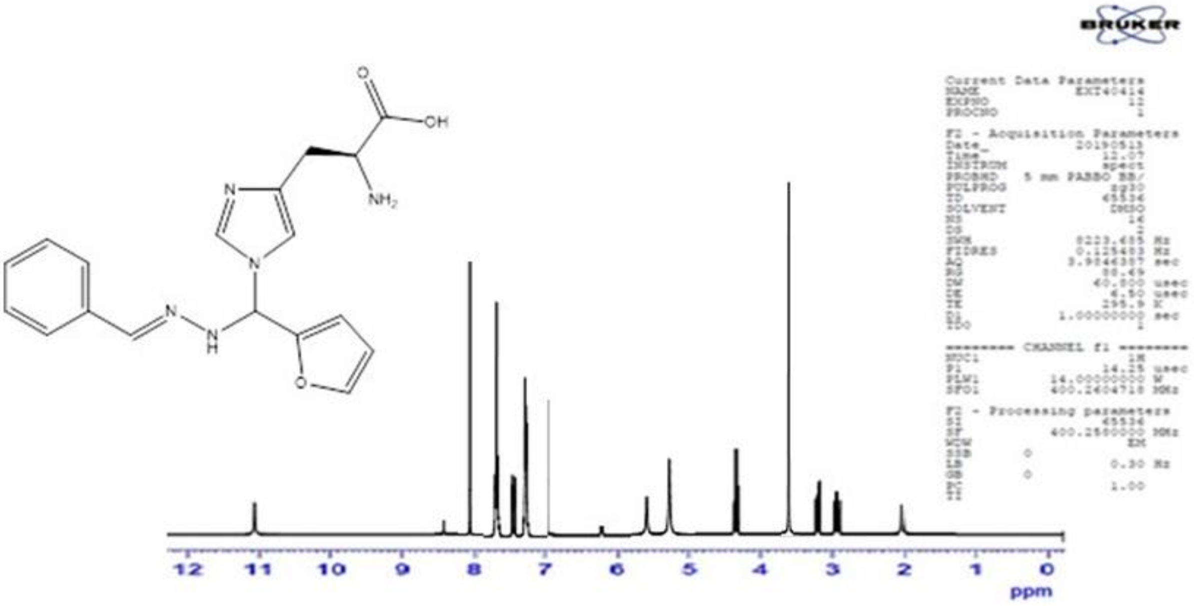

Figure S33 ^{1}H NMR spectrum of the compound **2c**

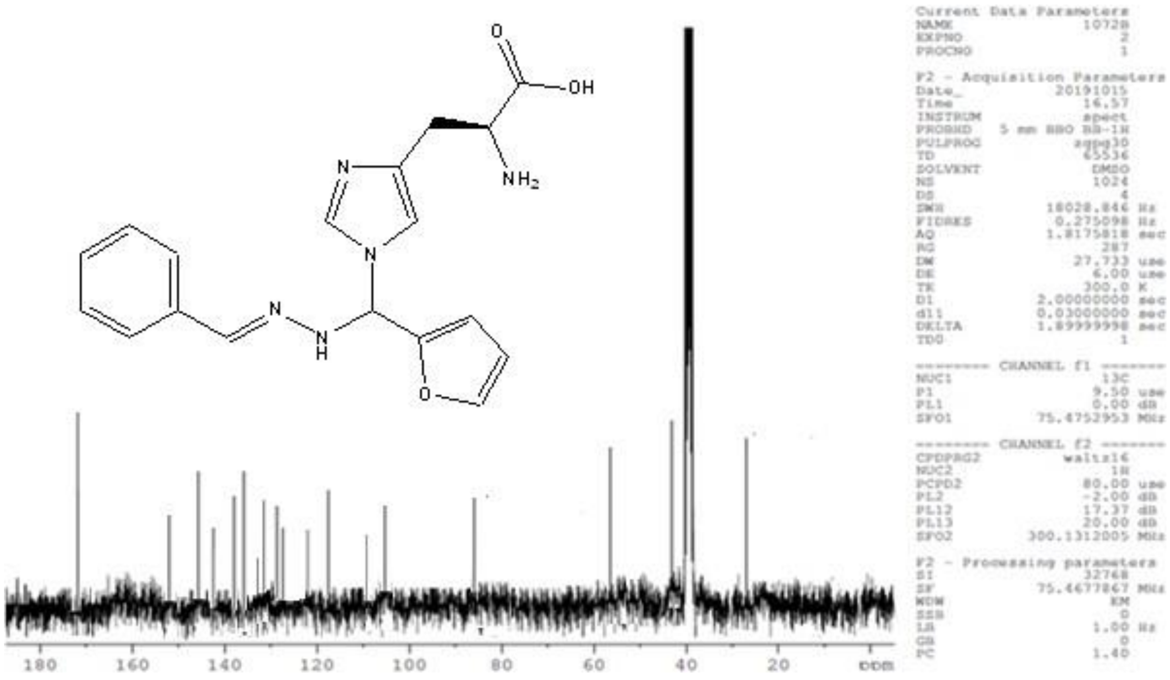

Figure S34 ^{13}C NMR spectrum of the compound **2c**

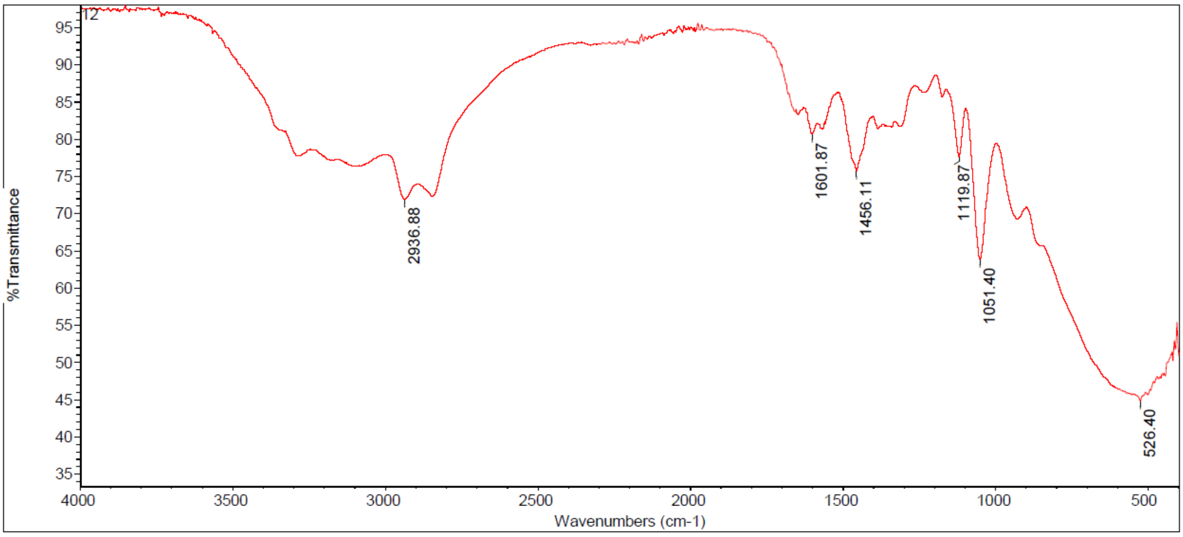

Figure S35 FTIR spectrum of the compound **2c**

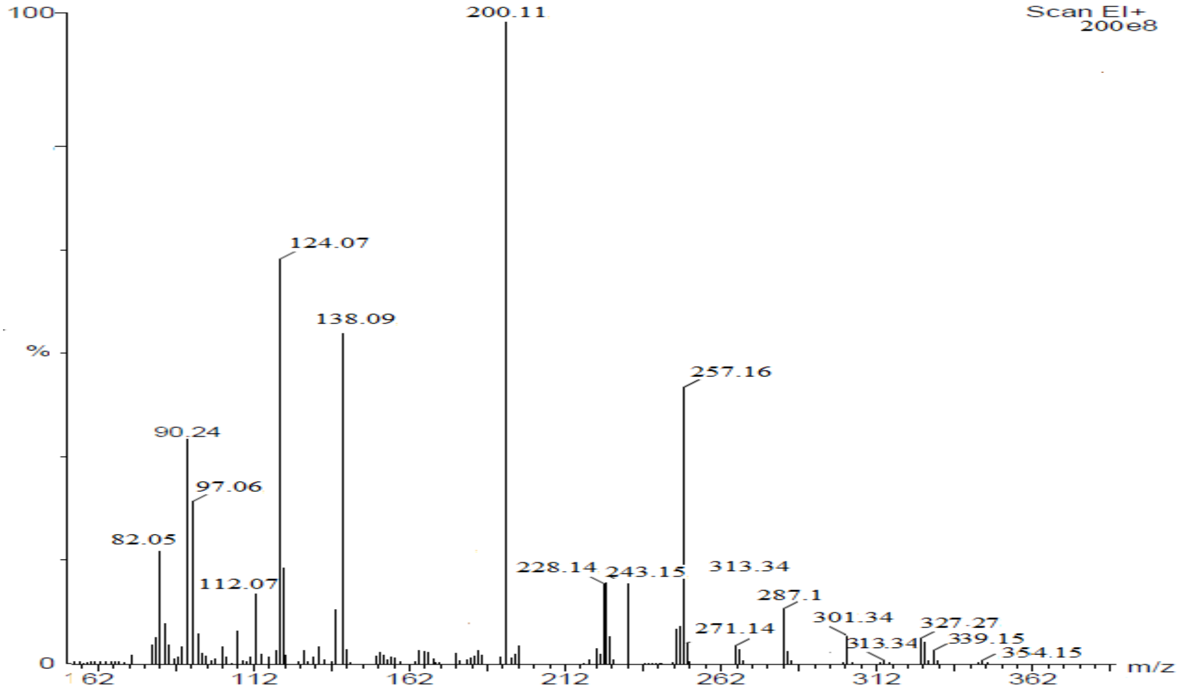

Figure S36 Mass spectrum of the compound **2c**

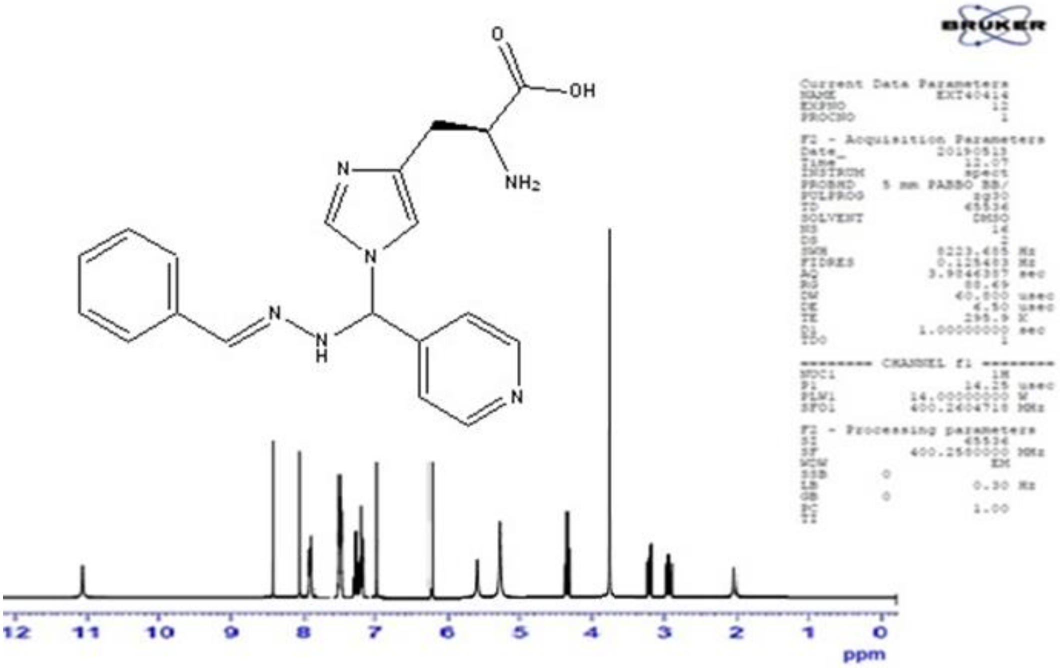

Figure S37 ^{1}H NMR spectrum of the compound **2d**

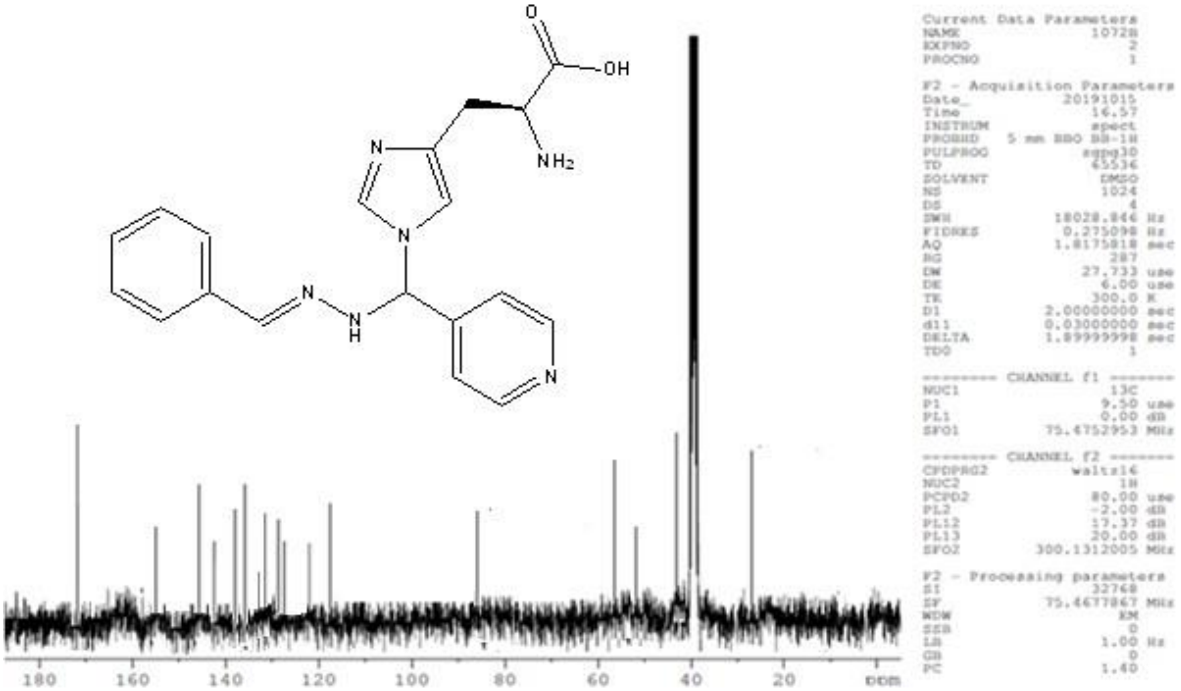

Figure S38 ^{13}C NMR spectrum of the compound **2d**

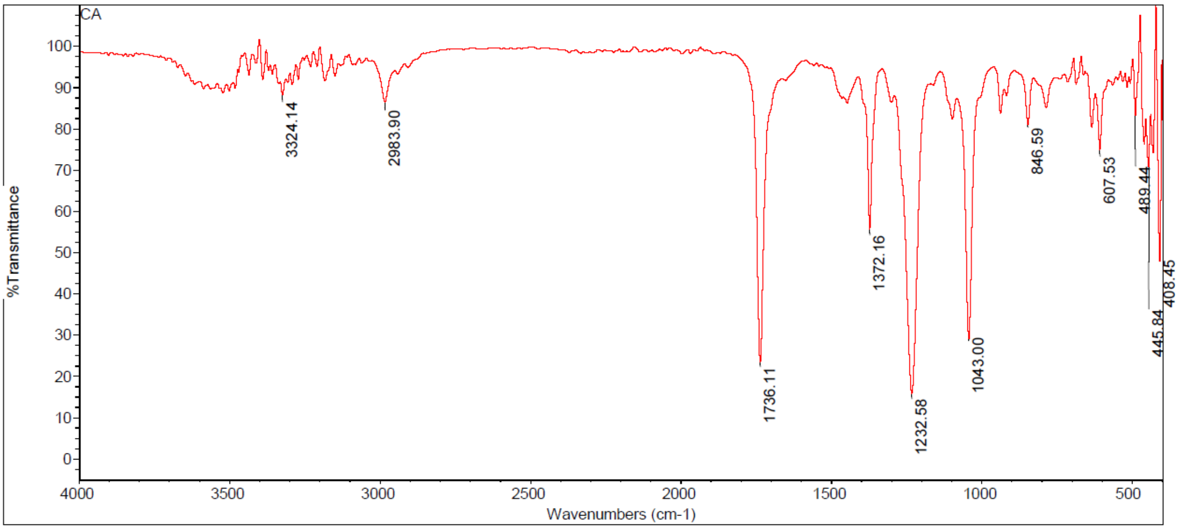

Figure S39 FTIR spectrum of the compound **2d**

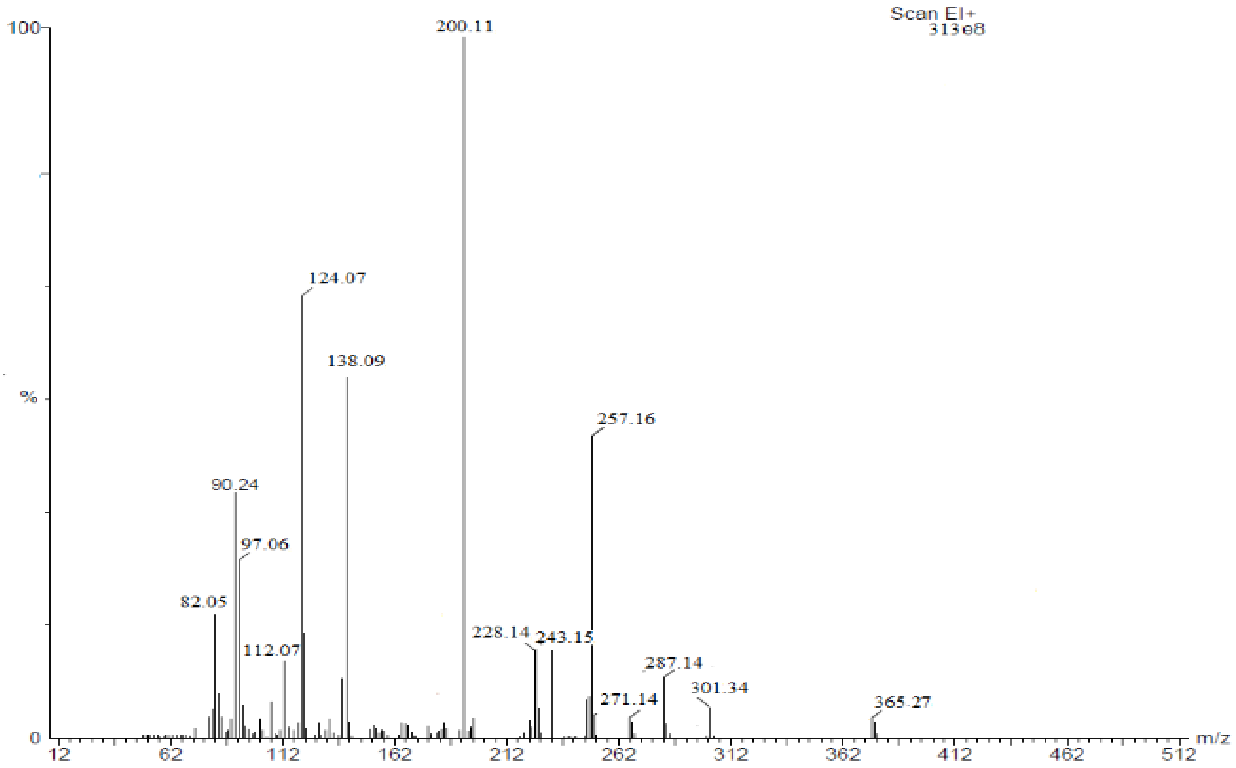

Figure S40 Mass spectrum of the compound **2d**

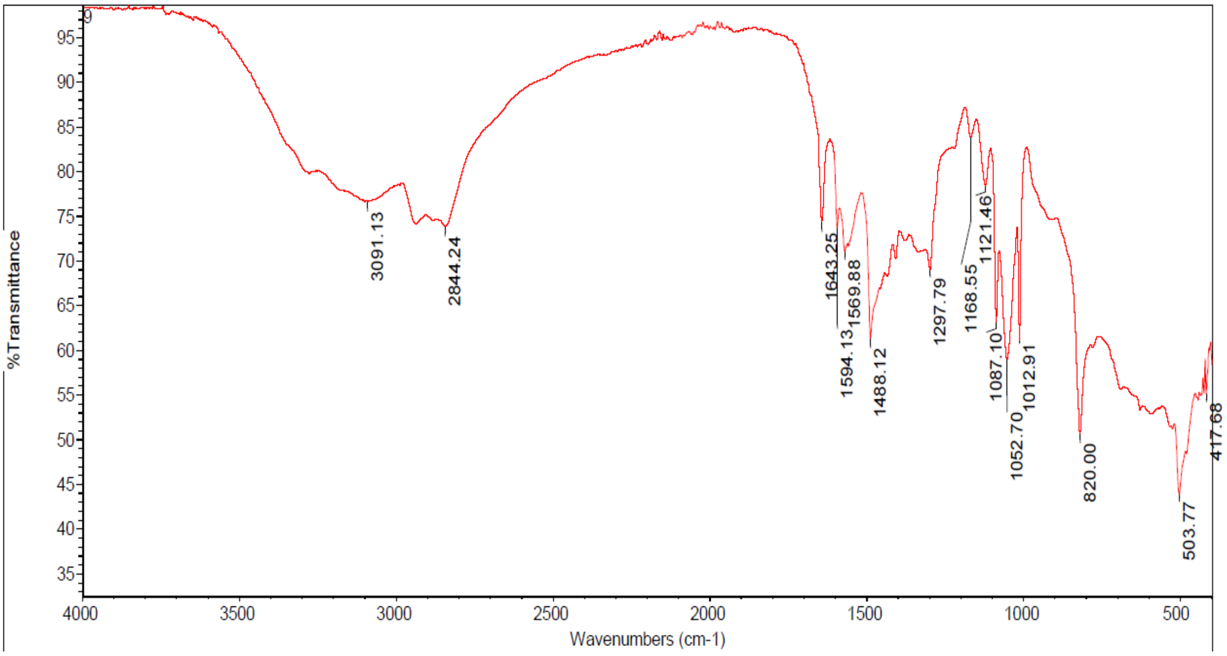

Figure S41 FTIR spectrum of the compound **2e**

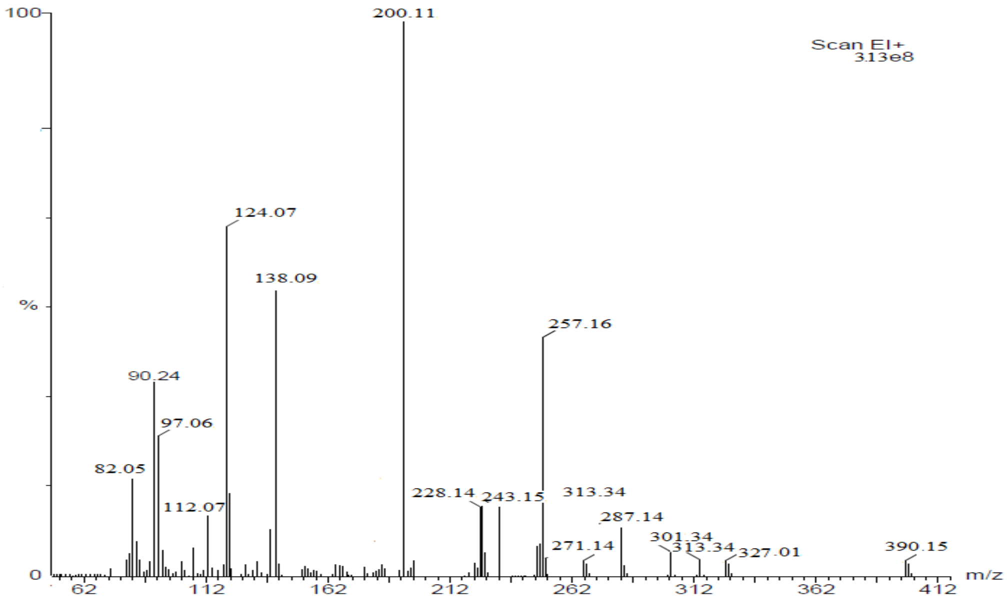

Figure S42 Mass spectrum of the compound **2e**

^{1}H spectra analysis tabulation of compound (1a-f) and (2a-e)

Table S1. ^{1}H spectra analysis tabulation of compound 1a

Chemical shift (ppm)	Multipllcity	Coupling Constant (Hz)	Integration
11.3	S	-	1H
8.36	S	-	1H
7.96	S	-	1H
7.02	S	-	1H
6.97	S	-	1H
5.11	S	-	2H
4.16	Dd	6Hz, 9Hz	1H
2.0	S	-	1H
1.12	M	-	10H
0.03	D	6Hz	2H

Table S2. ^{1}H spectra analysis tabulation of compound 1b

Chemical shift (ppm)	Multipllcity	Coupling Constant (Hz)	Integration
11.3	s	-	1H
8.36	s	-	1H
7.96	s	-	1H
7.02	s	-	1H
6.99	s	-	1H
5.35	s	-	1H
5.11	s	-	2H
4.16	dd	6Hz, 9Hz	1H
2.0	s	-	1H
1.12	m	-	5H
0.03	d	6Hz	2H
0.02	d	6Hz	4H

Table S3. ^{1}H spectra analysis tabulation of compound 1c

Chemical shift (ppm)	Multipllcity	Coupling Constant (Hz)	Integration
11.3	s	-	1H
8.36	s	-	1H
7.96	s	-	1H
7.02	s	-	1H
6.99	s	-	1H
5.11	s	-	2H
4.16	dd	6Hz, 9Hz	1H
2.0	s	-	1H
1.12	m	-	5H
0.03	d	6Hz	2H
0.02	d	6Hz	4H

Table S4. ^{1}H spectra analysis tabulation of compound 1d

Chemical shift (ppm)	Multipllcity	Coupling Constant (Hz)	Integration
11.3	s	-	1H
8.36	-	-	1H
7.96	s	-	1H
7.02	s	-	1H
6.99	s	-	1H
5.11	s	-	2H
4.16	dd	6Hz, 9Hz	1H
2.0	s	-	1H
1.12	m	-	5H
0.03	d	6Hz	2H
0.02	d	6Hz	4H

Table S5. ^{1}H spectra analysis tabulation of compound 1e

Chemical shift (ppm)	Multipllcity	Coupling Constant (Hz)	Integration
11.3	s	-	1H
8.36	s	-	1H
7.96	s	-	1H
7.02	s	-	1H
6.99	s	-	1H
5.11	s	-	2H
4.16	dd	6Hz, 9Hz	1H
3.83	s	-	3H
2.0	s	-	1H
1.12	m	-	5H
0.03	d	6Hz	2H
0.02	d	6Hz	4H

Table S6. ^{1}H spectra analysis tabulation of compound 1f

Chemical shift (ppm)	Multipllcity	Coupling Constant (Hz)	Integration
11.3	s	-	1H
8.36	s	-	1H
7.96	s	-	1H
7.02	s	-	1H
6.99	s	-	1H
5.11	s	-	2H
4.16	dd	6Hz, 9Hz	1H
3.06	s	-	6H
2.0	s	-	1H
1.12	m	-	5H
0.03	d	6Hz	2H
0.02	d	6Hz	4H

Table S7. ^{1}H spectra analysis tabulation of compound 2a

Chemical shift (ppm)	Multipllcity	Coupling Constant (Hz)	Integration
11.5	s	-	1H
8.36	s	-	1H
7.83	s	-	1H
6.88	s	-	1H
6.66	s	-	1H
5.33	s	-	1H
5.20	s	-	1H
5.11	s	-	2H
4.16	dd	6Hz, 9Hz	1H
2.18	s	-	2H
2.0	s	-	1H
1.98	s	-	2H
1.85	s	-	3H
1.82	s	-	3H
1.70	s	-	3H
1.55	s	-	2H
1.12	m	-	5H
0.03	d	6Hz	2H

Table S8. ^{1}H spectra analysis tabulation of compound 2b

Chemical shift (ppm)	Multipllcity	Coupling Constant (Hz)	Integration
11.5	s	-	1H
8.36	s	-	1H
7.83	s	-	1H
6.88	s	-	1H
6.66	s	-	1H
5.33	s	-	1H
5.11	s	-	2H
4.16	dd	6Hz, 9Hz	1H
2.0	s	-	1H
1.82	s	-	3H
1.70	s	-	3H
1.12	m	-	5H
0.03	d	6Hz	2H

Table S9. ^{1}H spectra analysis tabulation of compound 2c

Chemical shift (ppm)	Multipllcity	Coupling Constant (Hz)	Integration
11.5	s	-	1H
8.36	s	-	1H
7.83	s	-	1H
6.87	s	-	1H
6.66	s	-	1H
5.11	s	-	2H
4.16	dd	6Hz, 9Hz	1H
2.0	s	-	1H
1.12	m	-	5H
0.03	d	6Hz	2H
0.02	dd	6Hz, 9Hz	3H

Table S10. ^{1}H spectra analysis tabulation of compound 2d

Chemical shift (ppm)	Multipllcity	Coupling Constant (Hz)	Integration
11.3	s	-	1H
8.34	s	-	1H
7.83	s	-	1H
6.88	-	-	1H
6.66	s	-	1H
5.11	s	-	2H
4.16	dd	6Hz, 9Hz	1H
2.0	s	-	1H
0.31	m	-	5H
0.04	d	6Hz	4H
0.03	d	6Hz	2H

Table S11. ^{1}H spectra analysis tabulation of compound 2e

Chemical shift (ppm)	Multipllcity	Coupling Constant (Hz)	Integration
11.2	s	-	1H
8.36	s	-	1H
7.83	s	-	1H
6.88	s	-	1H
6.66	s	-	1H
5.11	s	-	2H
4.16	dd	6Hz, 9Hz	1H
2.0	s	-	1H
1.12	m	-	5H
0.37	s	-	2H
0.18	m	-	5H
0.03	d	6Hz	2H

^{13}C spectra analysis labeled compound (1a-f) and (1a-e)

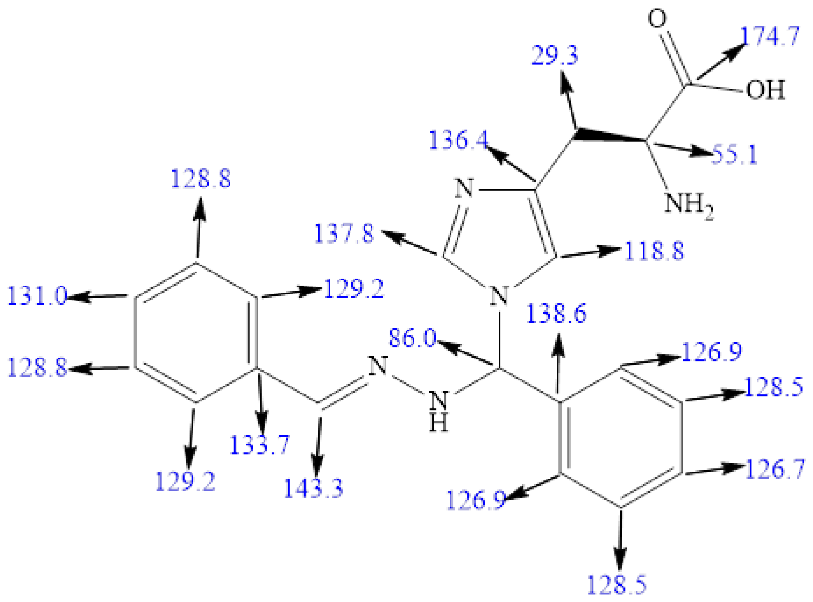

Figure S43^{13}C spectra analysis labeled compound 1a

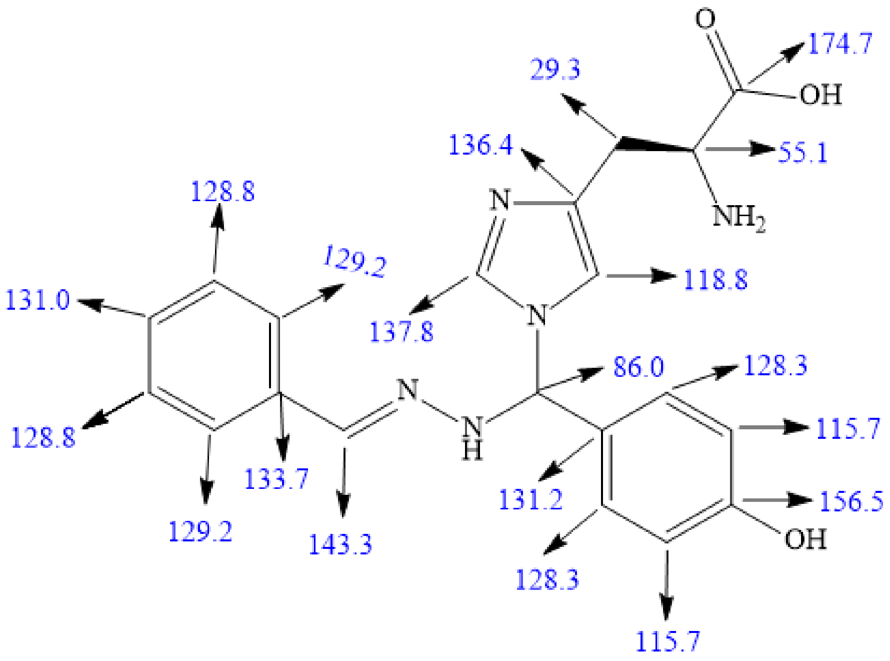

Figure S44 ^{13}C spectra analysis labeled compound 1b

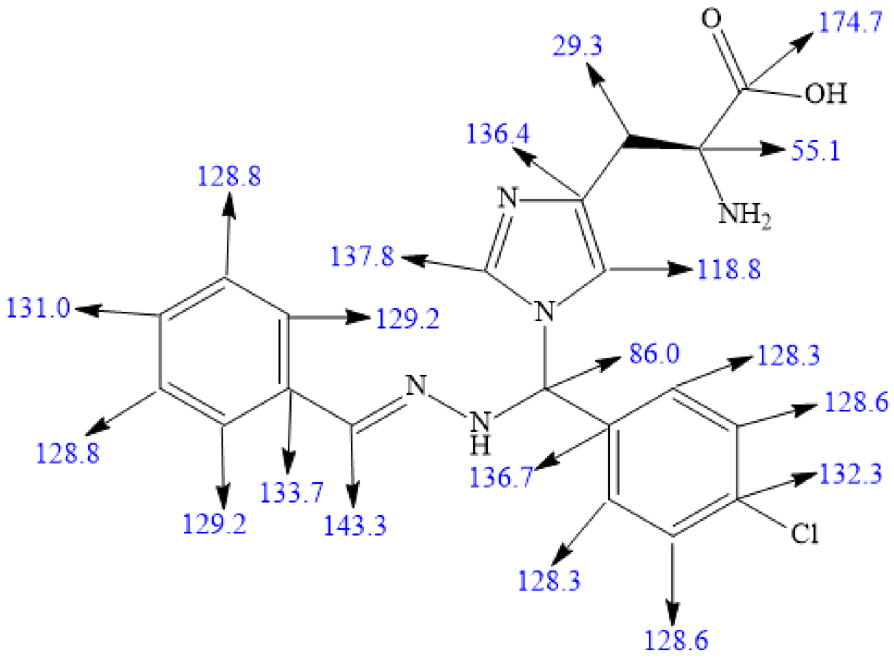

Figure S45 ^{13}C spectra analysis labeled compound 1c

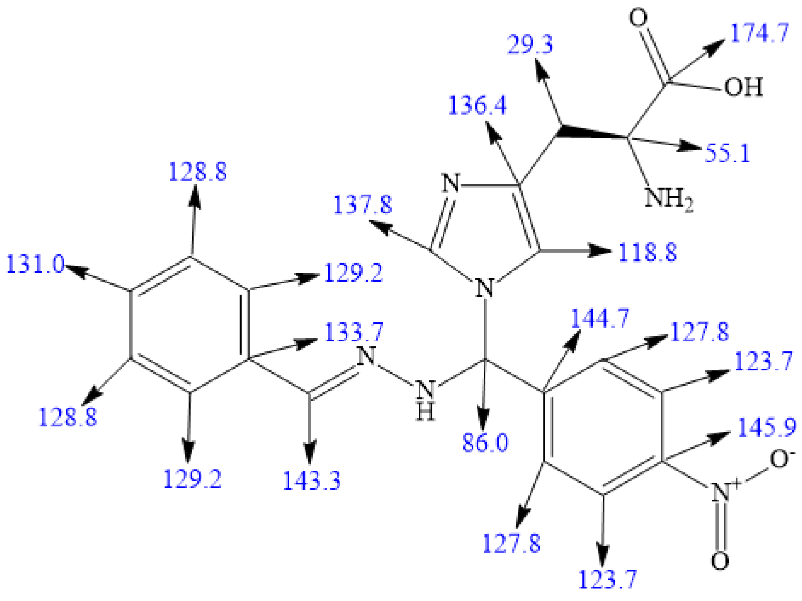

Figure S46 ^{13}C spectra analysis labeled compound 1d

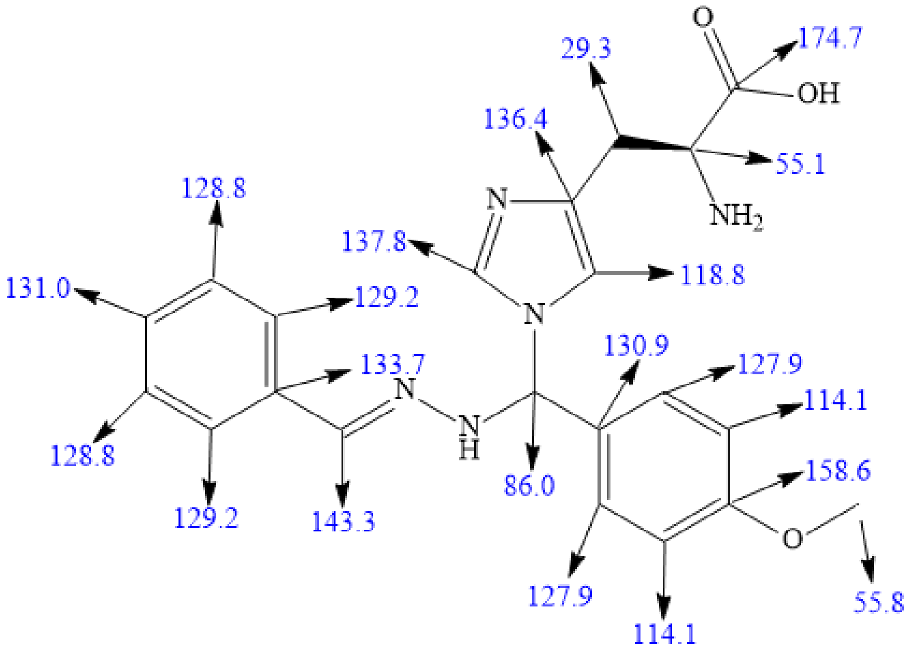

Figure S47 ^{13}C spectra analysis labeled compound 1e

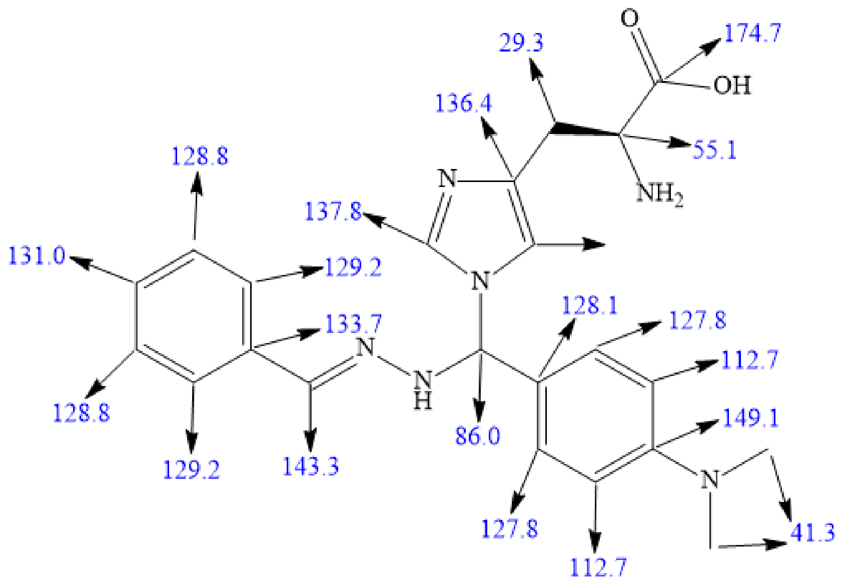

Figure S48 ^{13}C spectra analysis labeled compound 1f

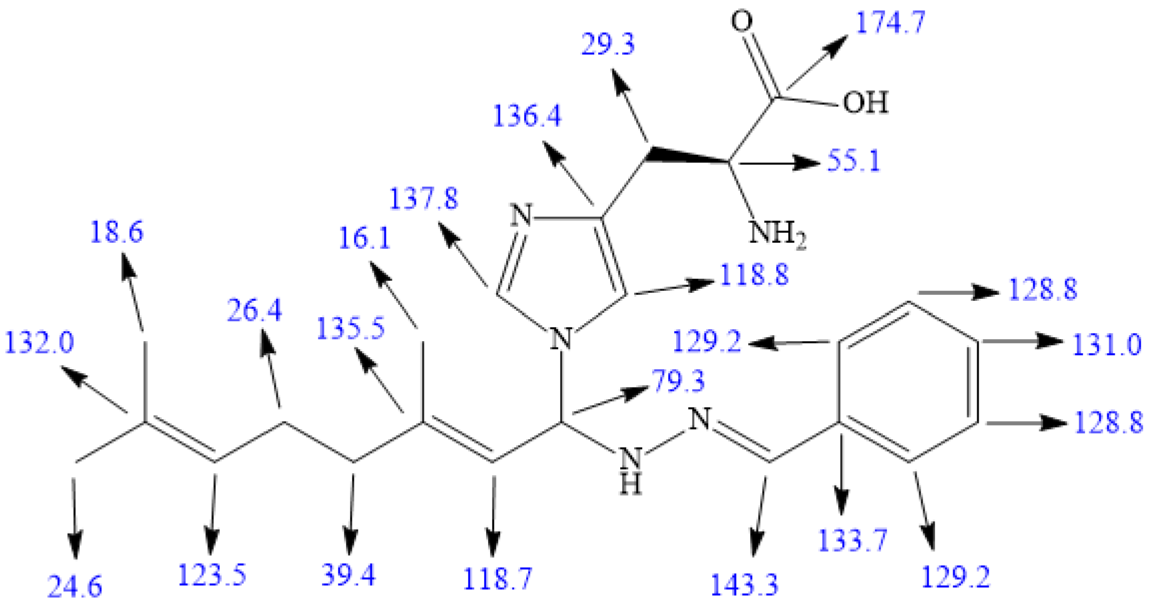

Figure S49 ^{13}C spectra analysis labeled compound 2a

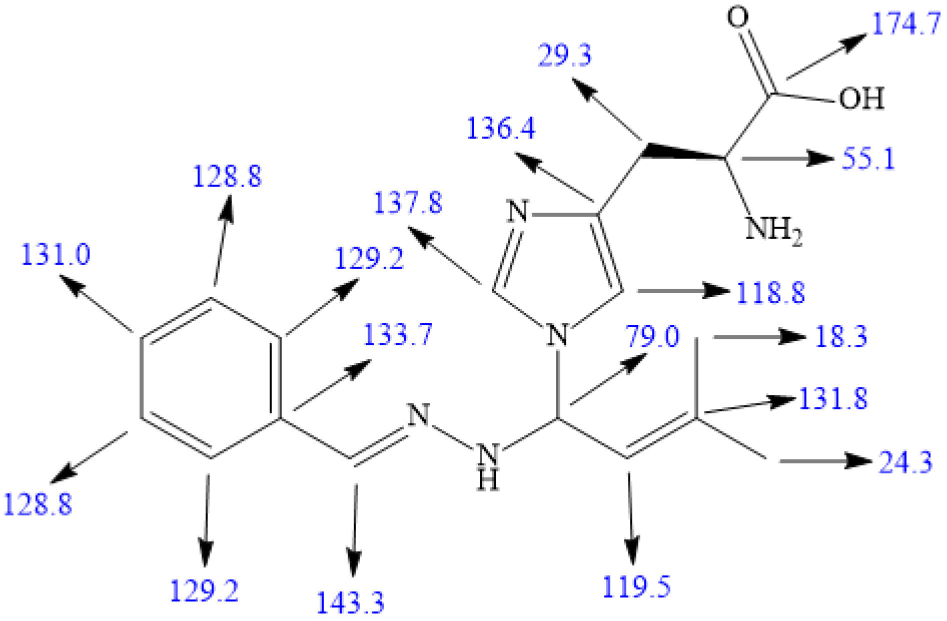

Figure S50 ^{13}C spectra analysis labeled compound 2b

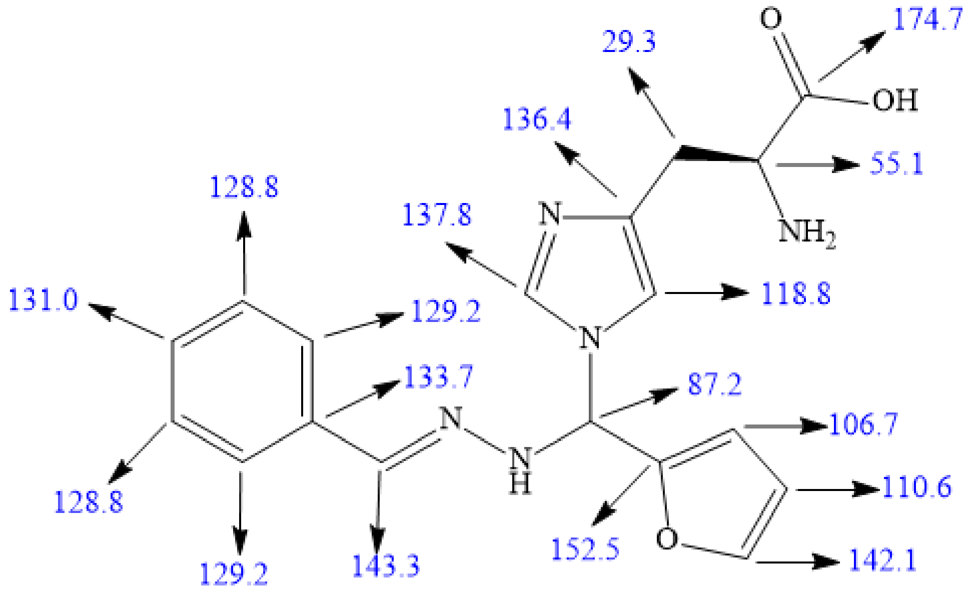

Figure S51 ^{13}C spectra analysis labeled compound 2c

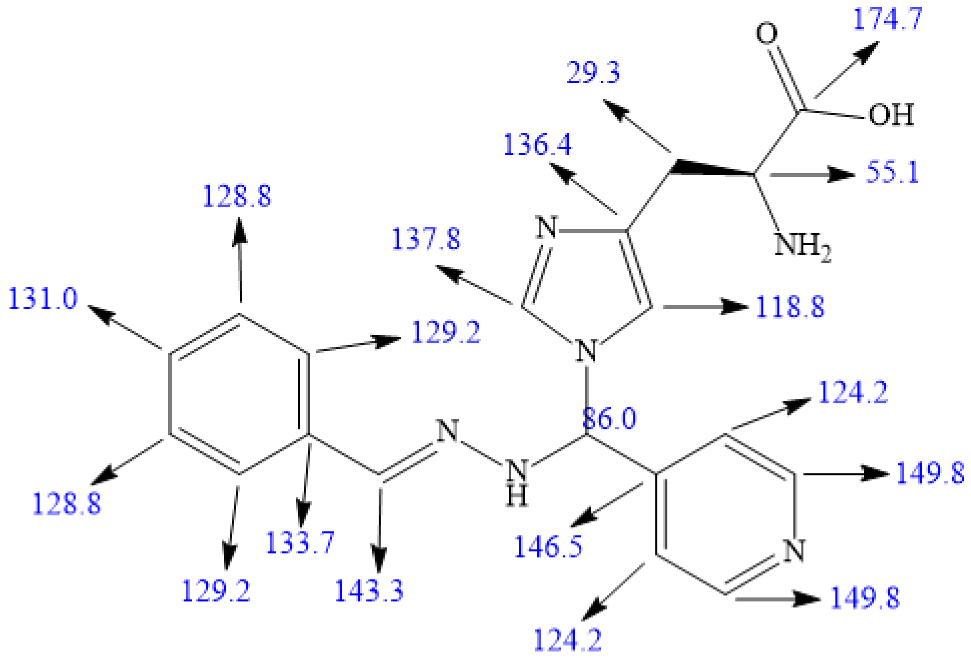

Figure S52 ^{13}C spectra analysis labeled compound 2d

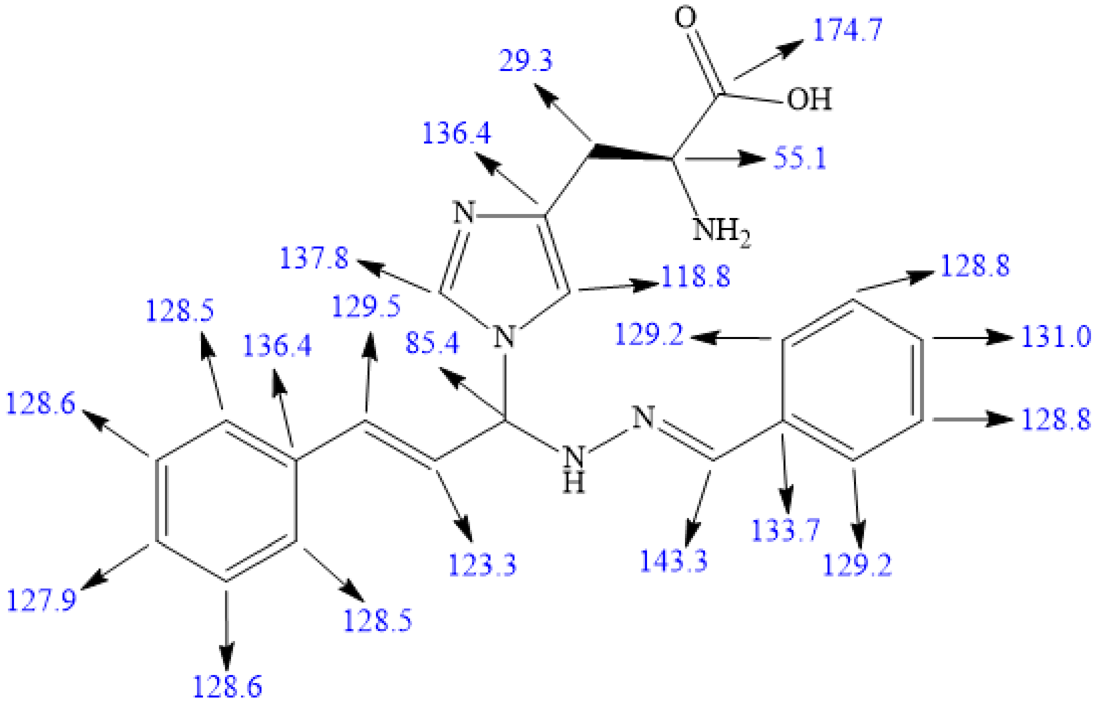

Figure S53 ^{13}C spectra analysis labeled compound 2e
